# Antisense molecules: A promising new therapy for atopic dermatitis

**DOI:** 10.1016/j.apsb.2025.09.008

**Published:** 2025-09-11

**Authors:** Jiayi Xue, Zhirong Yao

**Affiliations:** a Department of Dermatology, Xinhua Hospital, Shanghai Jiao Tong University School of Medicine, Shanghai 200092, China; b Institute of Dermatology, Shanghai Jiao Tong University School of Medicine, Shanghai 200092, China

**Keywords:** Antisense molecules, Atopic dermatitis, Gene therapy, RNA therapy, Disease modification, Clinical translations, Drug development, Preclinical testing, Clinical trials, Precision medicine

## Abstract

Atopic dermatitis (AD) is a common chronic inflammatory skin disorder affecting all age groups, especially children, with a prevalence of up to 20% globally. AD remains burdensome and incurable with current therapeutic strategies—ranging from trigger avoidance and skincare to medication—primarily address symptoms rather than disease modification, underscoring the imperative for innovative therapeutic paradigms. RNA-targeted therapies, particularly antisense molecules, have emerged as a transformative approach in precision medicine, with proven clinical success in diseases such as spinal muscular atrophy and familial chylomicronemia syndrome. These therapeutics achieve post-transcriptional regulation unattainable by conventional therapies, enabling direct targeting of messenger RNA (mRNA) and regulatory non-coding RNAs (ncRNAs) implicated in disease pathogenesis. Furthermore, skin is better suited to the antisense modulation due to the relatively easy access to target cells. Numerous studies have explored antisense-based targeting of key drivers in AD progression, yielding promising proof-of-concept results and prompting several early-stage clinical trials. This modality represents a paradigm shift in AD management—one that aligns with the broader revolution in RNA therapeutics reshaping modern medicine. This review critically examines the evolving role of antisense technology in AD, addressing both its mechanistic rationale and the translational challenges that must be overcome to realize its full clinical potential.

## Graphical abstract

Antisense molecule therapy offers a promising, novel approach targeting the molecular roots of atopic dermatitis at the RNA level, aiming for disease modification beyond current symptomatic treatments.

**Figure:**
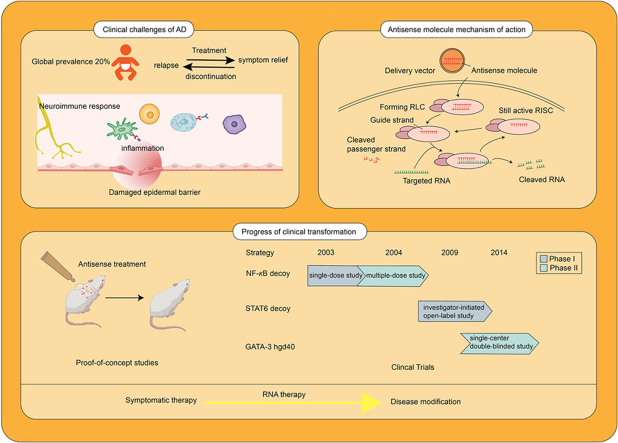

## 1 Introduction

Atopic dermatitis (AD), a chronic heterogeneous inflammatory skin disease, can affect up to 20% of children and 10% of adults worldwide, with over 200 million people impacted. Its prevalence has increased in both developed and developing nations in recent decades^1^. AD presents with a range of symptoms, from acute eczema-like eruptions such as erythema, papules, erosion, vesicles, and exudative lesions, to long-term skin thickening and lichenification^2^. The most disruptive symptom is intense, persistent itching, which not only interferes with daily life but also causes sleep disturbances^3^^,^^4^. Beyond skin issues, AD has multisystemic features, such as metabolic disease, including diabetes, cardiovascular disease, etc., as well as infections, malignancy, autoimmune diseases, and mental disorders^5^, ^6^, ^7^. Moreover, several studies indicate a positive correlation between the severity of AD and the incidence of these comorbidities^5^^,^^6^. AD is highly heritable, and its onset often triggers other allergic conditions like asthma, allergic rhinitis, and eosinophilic esophagitis, with the risk increasing with AD severity^8^, ^9^, ^10^. About a third of AD patients may eventually develop asthma, and another third will have allergic rhinitis^11^. As a lifelong condition, AD significantly impacts both patients and their families, reducing quality of life and placing a financial burden on society through rising healthcare costs and lost productivity^12^.

The pathogenesis of AD involves complex interactions between genetic factors, epidermal barrier dysfunction, immune system abnormalities, epigenetic changes, and so on^13^. Although treatment strategies for AD have evolved, challenges remain in addressing clinical needs. Traditional treatment regimens, primarily based on topical corticosteroids (TCS), topical calcineurin inhibitors, phosphodiesterase-4 inhibitors, phototherapy, and broad-spectrum immunosuppressive agents, have been complemented in recent years by the advent of biologics and small-molecule drugs^14^. Notably, dupilumab, targeting the interleukin (IL)-4/IL-13 receptor, nemolizumab, an IL-31 receptor inhibitor, and Janus kinase (JAK) inhibitors have improved outcomes for moderate-to-severe AD^15^, ^16^, ^17^. However, a substantial proportion of patients still experience treatment resistance or partial responses, and novel targeted therapies may be associated with adverse effects such as blepharitis, head and neck dermatitis, or exacerbation of acne^18^. While current therapies can achieve short-term symptom control, long-term management of AD faces dual challenges: first, traditional immunosuppressants may increase the risk of long-term complications such as leukopenia, hepatotoxicity, and nephrotoxicity due to systemic exposure^19^; second, existing targeted therapies fail to fully block the multi-pathway pathogenesis of AD, resulting in persistently high recurrence rates, which underscores the necessity of developing long-term therapies targeting the root causes of AD.

**Figure 1:**
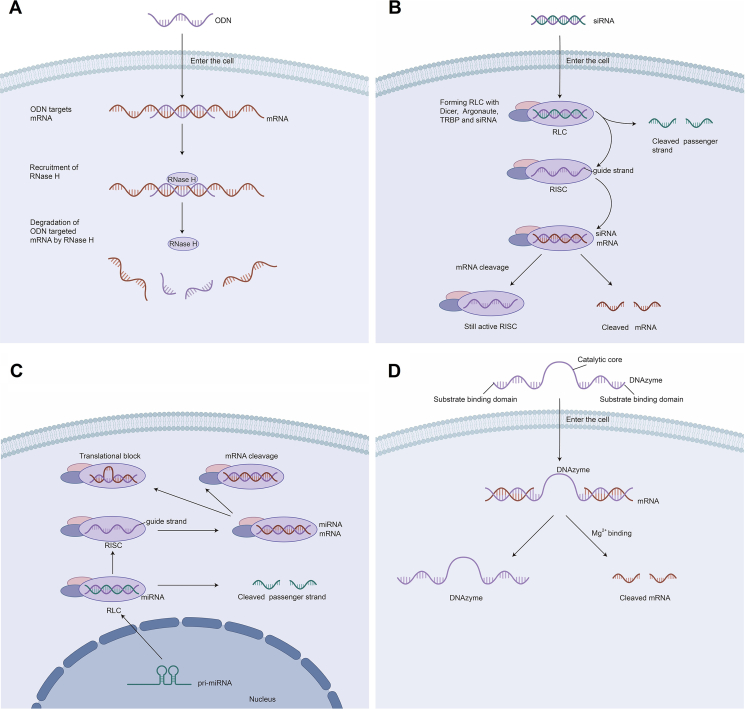
Mechanisms of oligonucleotide-induced regulation of gene expression. These four methods involve binding complementary oligonucleotides to the target RNA through base pairing, and thus all four methods essentially operate through an antisense mechanism. However, their downstream mechanisms differ significantly. (A) Antisense DNA (ODN) enter cells, bind to their target mRNA, and recruit ribonuclease H (RNase H), an enzyme that causes RNA cleavage. (B) *Via* endocytosis, siRNA enters the cytoplasm, where it combines with Dicer (RNase III endonuclease), Argonaute (RNase), and transactivation response element RNA-binding protein (TRBP) to form the RNA-induced silencing complex loading complex (RLC). To create a mature RNA-induced silencing complex (RISC), the RLC keeps the guide strand of siRNA and eliminates the passenger strand. Once the target mRNAs have sequences complementary to the siRNA guide strand, RISC may bind to them. Argonaute then induces cleavage of the mRNA and subsequent degradation by exonuclease. The RISC is a multiple-turnover enzyme, and thus the still-active RISC continues to bind to other target mRNA molecules. (C) In the nucleus, RNA polymerase II produce hairpin-containing pri mRNA, which is then processed by Drosha and di George critical region 8 proteins to form pre mRNA. Next, pre mRNA is exported to the cytoplasm, processed by Dicer into duplex, and loaded into the RLC for action. The mechanism of action is similar to that of siRNA. With the difference that the mature miRNAs are often not fully complementary to their targets and result in translational repression upon binding to the target mRNA. When a perfect match is made with the target, the miRNA exerts a cleavage effect. (D) The target RNA binds to the substrate binding domains of DNAzyme. After the recruitment of RNase H, the DNAzyme cleaves the target RNA in the presence of Mg^2+^.

Antisense molecule technology, as a focal point in contemporary biopharmaceutical research, offers a promising new direction^20^. They are a class of therapeutic oligonucleotides designed to target disease-associated messenger RNA (mRNA), or regulatory non-coding RNAs (ncRNAs). And after base complementary pairing, they modulate the gene expression in various ways^21^ (Fig. 1). Based on the broad-spectrum action characteristics of ncRNAs in gene regulation, antisense molecules can systematically target gene expression networks related to AD, achieving coordinated intervention of multi-pathway nodes^22^^,^^23^. This approach not only achieves long-term remission but also fundamentally alters the AD's course through epigenetic reprogramming^24^.

More and more antisense drugs have gained approval for various conditions, such as fomivirsen^25^, mipomersen^26^, nusinersen^27^, eteplirsen^28^, inotersen^29^, golodirsen^30^, volanesoren^31^, viltolarsen^32^, casimersen^33^, tofersen^34^, eplontersen^35^ (Table 1^25^, ^26^, ^27^, ^28^, ^29^, ^30^, ^31^, ^32^, ^33^, ^34^, ^35^, ^36^, ^37^, ^38^, ^39^, ^40^, ^41^). Several clinical trials are underway. CDR132L, a first-in-class microRNA (miRNA)-132 inhibitor, was safe and well-tolerated in a Phase 1b trial for chronic ischemic heart failure^42^. A Phase IIa clinical trial of BP1001, an antisense DNA (ODN) targeting growth factor receptor-bound protein 2, in combination with venetoclax and decitabine in patients with acute myeloid leukemia who are not candidates for intensive induction therapy is ongoing, with interim data showing encouraging efficacy and no drug-related toxicity (NCT02781883)^43^. Bepirovirsen targeting all hepatitis B virus (HBV) mRNA was shown to continuously reduce hepatitis B surface antigen and HBV DNA in 9 to 10% of patients with chronic HBV infection in a phase 2b trial^44^ (Table 2^42^, ^43^, ^44^). These examples highlight the potential of antisense therapies (Table 3^21^, ^22^, ^23^, ^24^^,^^45^, ^46^, ^47^, ^48^, ^49^, ^50^, ^51^, ^52^ provides a brief overview of different kinds of antisense molecules) as a novel and feasible therapeutic strategy for diseases like AD. Additionally, since the skin is accessible, antisense drugs can act directly on the lesion without the need for liver metabolism, which has a unique advantage for treating skin diseases^53^^,^^54^. To become a reality for treating AD, however, therapeutic antisense strategy in AD is still in its infancy and must first clear some significant obstacles. This review focuses on the latest advances and ongoing efforts to bring antisense therapeutics to AD.

**Table 1:** Antisense therapeutics approved by the FDA and EMA.

Drugs name	Target	Indication	Administration	Approval year	Ref.
ODN					
Fomivirsen	*CMV IE2*	Cytomegalovirus retinitis	Intravitreal	1998^a^^2002^	25
Mipomersen	Apolipoprotein B-100	Hypercholesterolemia	Subcutaneous	2013^a^^2018^	26
Nusinersen	*SMN2*	Spinal muscular atrophy	Intrathecal	2016	27
Eteplirsen	Dystrophin gene exon 51	Duchenne muscular dystrophy	Intravenous	2016	28
Inotersen	*TTR*	Hereditary transthyroxine amyloidosis	Subcutaneous	2018	29
Golodirsen	Dystrophin gene exon 53	Duchenne muscular dystrophy	Intravenous	2019	30
Volanesorsen	Apolipoprotein CIII	Familial chylomicronemia syndrome	Subcutaneous	2019	31
Viltolarsen	Dystrophin gene exon 53	Duchenne muscular dystrophy	Intravenous	2020	32
Casimersen	Dystrophin gene exon 45	Duchenne muscular dystrophy	Intravenous	2021	33
Tofersen	Superoxide dismutase 1	Amyotrophic lateral sclerosis	Intrathecal	2023	34
Eplontersen	*TTR*	TTR-mediated amyloidosis	Subcutaneous	2023	35
siRNA					
Givosiran	Delta-ALA synthase 1	Acute hepatic porphyria	Subcutaneous	2019	36
Lumasiran	*HAO1*	Primary hyperoxaluria type 1	Subcutaneous	2020	37
Inclisiran	*PCSK9*	Primary hypercholesterolemia/mixed dyslipidemia in adults	Subcutaneous	2020	38
Vutrisiran	*TTR*	hATTR-PN	Subcutaneous	2022	39
Nedosiran	*LDHA*	Primary hyperoxaluria type 1	Subcutaneous	2023	40
Patisiran	*TTR*	TTR-mediated amyloidosis	Intravenous	2018	41

hATTR-PN, hereditary transthyretin amyloid polyneuropathy; LDHA, lactate dehydrogenase A in hepatocytes, ALA, aminolevulinic acid.

a Withdrawal of approval.

**Table 2:** The current preclinical antisense drugs (mentioned in the text).

Drugs name	Target	Indication	Administration	Phase	Ref.
miRNA antagonist					
CDR132L	miR-132	Chronic ischemic heart failure	Intravenous	Phase 1b	42
ODN					
BP1001	*Grb2*	Acute myeloid leukemia	Intravenous	Phase 2	43
Bepirovirsen	HBV mRNA	Chronic HBV infection	Subcutaneous	Phase 2b	44

HBV, hepatitis B virus.

**Table 3:** Characteristics of major antisense molecule families^21^, ^22^, ^23^, ^24^^,^^39^, ^40^, ^41^, ^42^, ^43^, ^44^, ^45^, ^46^.

Antisense molecule	Antisense DNA (ODN)	siRNA	miRNA	DNAzyme
Structure	Linear ssDNA or dsDNA	Linear dsRNA	Linear ssRNA	ssDNA with two binding domains encircling a central catalytic domain
Length	15–20 nucleotides	21–25 nucleotides	19–25 nucleotides	30–35 nucleotides
Enzymes needed	Ribonucleases H1	RNA-induced silencing complex	No	No
Modifications	*e*.*g*., 2′-*O*-Me, 2′-MOE and locked nucleic acids	*e*.*g*., 3′-inverted thymidine	*e*.*g*., 2′-F, 2′-*O*-Me and 2′-MOE	*e*.*g*., 3′-inverted thymidine and PS
Advantages	High target affinity Extensive modification capacity → improved nuclease resistance and pharmacokinetics	Endogenously processed by Dicer Complete mRNA degradation	Multi-gene regulation Extensive modification capacity → enhanced drug properties	Low immunogenicity Enhanced cell penetration Ease of synthesis Minimal side effects
Disadvantages	Off-target effects Chemistry-dependent toxicity	Poor *in vivo* stability Inadequate cell uptake	Low specificity Unforeseen side effects	Mg^2+^-dependent

ss, single-stranded; ds, double-stranded; 2′-*O*-Me, 2′-*O*-methyl; 2′-MOE, 2′-*O*-methoxymethyl; 2′-F, 2′-fluoro; PS, phosphorothioate.

## 2 Causes and molecular pathogenesis of AD

### 2.1 Genetic defects

Typically, AD patients have a family history of atopic diseases. To date, a variety of AD susceptibility genes have been identified; the most important ones are those encoding structural and functional proteins of the epidermis and proteins that modulate the innate and adaptive immune responses^55^. One key risk factor for AD is epithelial barrier dysfunction caused by mutations in the filaggrin (*FLG*) gene^56^. *FLG*, a major protein in the stratum corneum (SC), aggregates keratin fibers to form a barrier that prevents moisture loss and protects against allergens^57^. Deamination of the *FLG* produces a natural moisturizing factor, which can retain an adequate amount of moisture in the skin, maintain the skin pH value at a proper level, and then inhibit the expansion of *Staphylococcus aureus*^58^. The *FLG* loss-of-function mutation will impair the skin barrier, leading to increased water loss, elevated skin pH, and greater permeability to allergens, which heighten the risk of AD^59^^,^^60^. Additionally, roughly half of patients with moderate to severe AD had *FLG* with null mutations^61^. Genetic barrier defects also reduce the expression of other key structural proteins, such as keratin, loricrin, involucrin, and cladin-1, claudin-23, compromising the mechanical barrier function of the skin^62^. In addition, a European and multi-ancestry genome-wide association meta-analysis of AD reported two other genes significantly associated with AD. They were *S100A9* (OR = 1.36, *P* = 5.5E-212) and *RORC* (OR = 1.24, *P* = 3.1E-134), whereas the association of *FLG* with AD had an OR = 1.41, *P* = 1.4E-228^63^. *RORC*, encoding the Th17-specific transcription factor retinoid-related orphan nuclear receptor gamma t, drives Th17 differentiation and synergizes with other factors to activate Th17-associated genes (*e*.*g*., *IL17A*, *IL17F*, and *IL23R*)^64^. Th17 cells, which are closely associated with AD's pathogenesis, produce cytokines like IL-17A, IL-17F, and IL-23. These cytokines are elevated in pediatric AD patients and positively correlate with disease severity^65^. In murine models of AD, IL-17E upregulation stimulates the production of endothelin-1, a key pruritogenic mediator^66^. Studies have demonstrated that IL-17 can induce the expression of antimicrobial peptides (*e*.*g*., IL-36*γ*, S100A7, and human beta-defensin 2), chemokines including C–X–C motif chemokine ligand 8 and C–C motif ligand 5 (CCL5), as well as other cytokines like vascular endothelial growth factor, IL-8, and granulocyte-macrophage colony-stimulating factor, while also promoting Th2 immune responses^67^^,^^68^. The Th17/Th22 pathway can induce the expression of S100A9, which is highly elevated in AD patients' serum and skin tissues, correlating strongly with the disease severity^69^. Extracellular S100A9 acts as a pro-inflammatory “sirens”, promoting neutrophil, monocyte, and lymphocyte chemotaxis *via* Toll-like receptor 4 (TLR4) and enhancing Th2 responses by inducing keratinocyte-derived IL-33 through the receptor of advanced glycation endproducts^70^^,^^71^. However, no study has yet revealed a direct link between mutations in either of these genes and the development of AD.

### 2.2 Immune dysregulation

**Figure 2:**
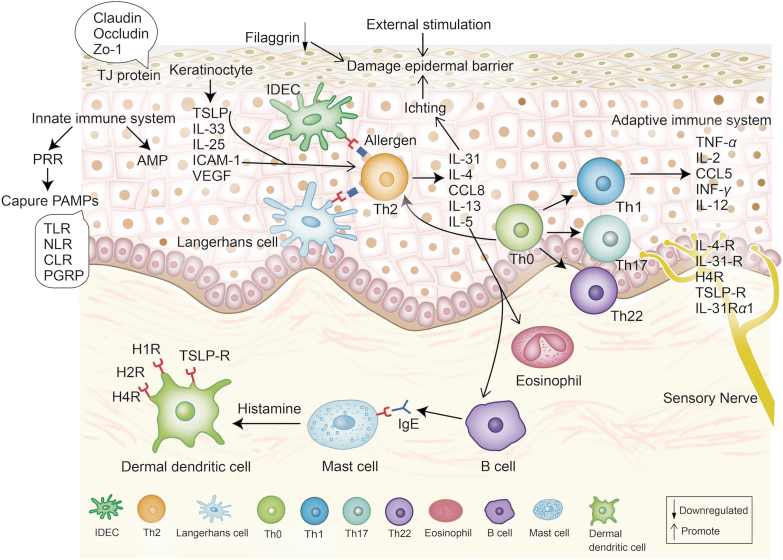
Immunological dysregulation of AD. Zo-1, zonula occludens protein-1; TJ, protein tight junction protein; PRR, pattern recognition receptors; AMP, antimicrobial peptides; PAMPs, pathogen-associated molecular patterns; TLR, Toll-like receptors; NLR, NOD-like receptors; PGRP, peptidoglycan recognition protein; TSLP-R, thymic stromal lymphopoietin receptor; H1R, histamine H1 receptor; VEGF, vascular endothelial growth factor; IDEC, inflammatory dermal dendritic cell; INF-*γ*, interferon gamma; TNF-*α*, tumor necrosis factor alpha.

Immunologic factors are also important in the development of AD (Fig. 2). One theory suggests that impaired skin barriers lead to immune dysregulation, while another posits that immune abnormalities make the skin more vulnerable to environmental stimuli^72^. Anyway, targeting the immune system is crucial in the treatment of AD. The main immunological features of AD are Th2 cell activation and related cytokine expression (*e*.*g*., IL-4, IL-5, IL-13, and IL-31), further promoting B cell proliferation, IgE synthesis, and eosinophilic activation^13^. The activation of T cells is regulated by multi-layered signaling mechanisms. Following antigen recognition by the T cell receptor, which generates the first signal, the downstream signal molecule, such as IL-2-inducible tyrosine kinase, amplifies the signaling cascade within T cells^73^^,^^74^. The second signal for T cell activation is provided by the interaction between CD86 on antigen-presenting cells and CD28 on T cells^75^^,^^76^. IL-4 and IL-13 work by binding to IL-4 receptor type II, triggering the phosphorylation of JAK1 and tyrosine kinase 2 (TYK2), leading to the activation of signal transducer and activator of transcription 3 (STAT3) and STAT6, which enter the nucleus to initiate AD cytokine-related gene expression^77^. Additionally, aberrantly expressed IL-10 in AD lesions activates the STAT1/STAT3 signaling axis *via* JAK1/TYK2, playing a pivotal role in regulating Th17/Th22 immune responses, which are more prominent in chronic AD. And these two signaling pathways can be inhibited by cytokine signaling transmission1 (SOCS1) inhibitors^78^.

Beyond the adaptive immune system, the innate immune system, serving as the skin's first line of defense, also plays a significant role in the development of AD. Keratinocytes, the primary cellular component of the epidermis, are key players in the skin's innate immunity^79^. Upon exposure to external environmental stimuli, the epidermis is damaged, and keratinocytes secrete pro-inflammatory cytokines such as thymic stromal lymphopoietin (TSLP), IL-33, IL-25, and intercellular adhesion molecule-1 (ICAM-1), which promote the maturation and migration of eosinophils, Th2 cells, and other immune cells^80^^,^^81^. Furthermore, macrophage-derived chemokine CCL22 is significantly elevated in the serum of AD patients and correlates with disease severity, helping recruit Th2 lymphocytes^82^, ^83^, ^84^. Pattern recognition receptors, which are mainly expressed on the surface of innate immune cells, identify pathogen-associated molecular patterns and help to trigger innate immunity^85^^,^^86^. Pattern recognition receptors related to AD mainly include TLRs, NOD-like receptors (NLRs), C-type lectin receptors, etc^87^. TLRs and NLRs are the main receptors against *S. aureus*, and damage to these receptors increases *S. aureus* colonization, which is common in AD^88^. The adaptive immune response also regulates the innate immune system in AD, for instance, under the action of Th2 cytokines, antimicrobial peptides are suppressed, and dendritic cells also lose their ability to release anti-inflammatory IL-10 against bacteria^89^, ^90^, ^91^. Therefore, therapies targeting the Th2 bias in AD may help restore balance to both the adaptive and innate immune responses.

### 2.3 Damaged epidermal barrier

AD often originates from skin barrier dysfunction, which is driven by the synergistic interaction between genetic defects (as previously mentioned), environmental factors, and inflammation, creating a vicious cycle of “barrier damage-environmental stimulus-immune activation-barrier deterioration”^92^^,^^93^. In AD patients, ceramide levels are significantly reduced, correlating positively with disease severity^94^. The activities of proteases secreted by *S. aureus* and house dust mites are markedly elevated in AD lesions, while the activity of endogenous protease inhibitors is insufficient, resulting in increased barrier permeability and amplified inflammation. In addition, the use of soap or detergent increases skin pH, together with the activation of serine protease, which accelerates the degradation of ceramide synthetase, leading to disorganized lipid layer structures and further compromising the hydrophobic barrier function^95^. *In vitro* studies show that elevated Th2 cytokines can induce barrier defects even without genetic mutations by downregulating key epidermal proteins like FLG, loricrin, and involucrin, thus impairing barrier function^96^. Notably, pruritus-related inflammatory cytokines such as IL-31 induce scratching behavior, further exacerbating physical barrier damage^97^.

### 2.4 Epigenetic changes

The rapid rise in the prevalence of AD in recent years cannot be fully explained by the low probability of genetic mutations alone. Instead, environmental factors, such as exposure to house dust mite allergens, have emerged as significant contributors to AD development^98^. Epigenetic mechanisms, including histone modifications, DNA methylation, and miRNA dysregulation, have been identified as key mediators of environmental influences on gene expression. Histone modifications such as acetylation induce tighter chromatin packing, thereby suppressing transcription, while deacetylation promotes chromatin relaxation and transcriptional activation. DNA methylation targets cytosine-phosphate-guanosine (CpG)-rich promoter sequences and inhibits gene expression by blocking transcription factor binding^55^^,^^99^.

**Figure 3:**
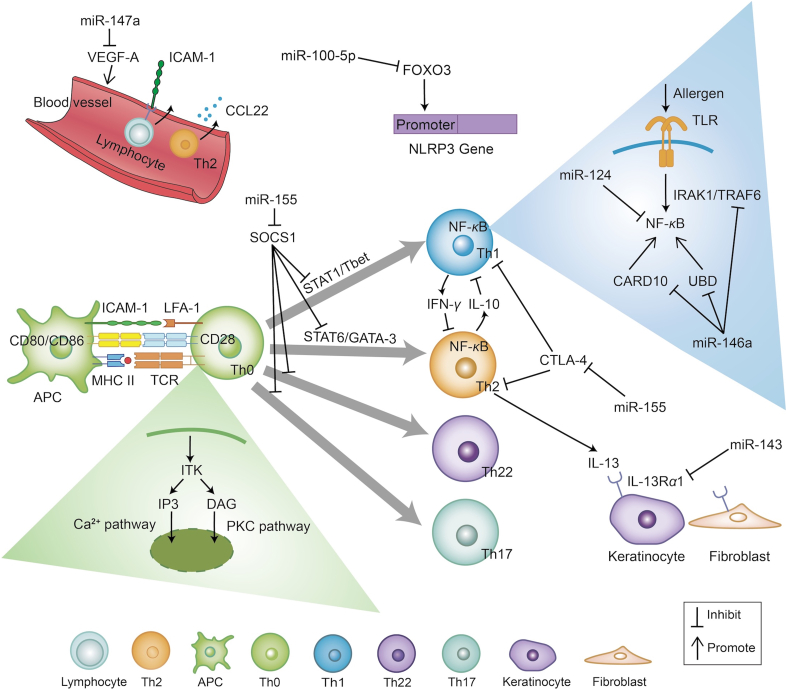
Antisense therapies based on the pathogenesis of AD. APC, antigen-presenting cells; ICAM-1, intercellular adhesion molecule-1; LFA-1, leukocyte function-associated antigen-1; MHC II, major histocompatibility complex Ⅱ; TCR T, cell receptor; ITK, IL-2-inducible tyrosine kinase; IP3, inositol triphosphate; DAG, diacylglycerol; PKC, protein kinase C; SOCS1, suppressors of cytokine signaling 1; STAT1, signal transducers and activators of transcription 1; T-bet, T-box expressed in T cells; GATA-3, guanine adenine thymine adenine sequence-binding protein 3; NF-*κ*B, nuclear factor kappa-B; CTLA-4, cytotoxic T lymphocyte-associated antigen-4; CARD10, caspase recruitment domain-containing protein 10; IRAK1, IL-1 receptor–associated kinase 1; UBD, ubiquitin D; TRAF6, tumor necrosis factor receptor-associated factor 6; CCL22, chemokine ligand 22; VEGF-A, vascular endothelial growth factor-A.

The interaction between miRNA therapy and important targets in the pathogenesis of AD has been extensively studied (Fig. 3). Sonkoly et al.^100^ and Yao et al.^101^ identified 44 differentially expressed miRNAs (34 downregulated and 10 upregulated) in AD lesional skin compared to healthy controls. Notably, miRNA-155, one of the most upregulated miRNAs in AD, positively correlates with disease severity. miR-155 can activate the JAK/STAT pathway by targeting SOCS1 and exacerbate T cell activation by inhibiting cytotoxic T lymphocyte-associated antigen-4 expression^100^^,^^101^. One study confirmed that miR-155-5p destroyed tight junction (TJ) protein by targeting protein kinase inhibitor^102^. Conversely, miR-143 is downregulated in AD lesions and directly targets *IL13RA1* mRNA, regulating the impact of IL-13 on epidermal barrier function^100^^,^^103^. Another miRNA, miR-100-5p, inhibits the NLRP3 (NLR family, pyrin domain containing 3) signaling pathway by targeting forkhead box protein 3 (a transcription factor of the O subclass of the forkhead family), thereby reducing inflammation and pyroptosis^104^. Additionally, miR-146a, known for its role in innate immune responses, suppresses the expression of immune-related factors in keratinocytes, such as IL-1 receptor-associated kinase 1 and tumor necrosis factor (TNF) receptor-associated factor 6, both of which are critical molecules in TLR signaling^105^. Rebane et al.^106^ further demonstrated elevated miR-146a levels in chronic AD lesions and identified novel targets, including CCL5 and caspase recruitment domain-containing protein 10, which mediate the degradation of nuclear factor kappa-B (NF-*κ*B) inhibitors. Yang et al.^107^ reported reduced miR-124 expression in AD lesions, inversely correlated with elevated levels of *RELA*, *IL8*, *CCL5*, and *CCL8* mRNA. Recently, a study found that miR-147a is significantly downregulated in the serum and skin lesions of AD mice^108^.

Furthermore, prenatal and postnatal environmental exposures can permanently alter the epigenome of offspring^109^^,^^110^. For example, maternal tobacco exposure during pregnancy increases miR-223 expression in umbilical cord blood, reduces methylation levels at the TSLP 5′CpG island, and alters DNA methylation patterns at the forkhead box protein 3 locus, leading to reduced Treg cell counts and heightened susceptibility to AD within the first three years of life^99^^,^^111^^,^^112^.

### 2.5 Other aspects

Pruritus, one of the hallmark symptoms of AD, arises from a highly organized interplay among keratinocytes, the immune system, and the nervous system. Chronic itch in AD is primarily driven by non-histaminergic pathways^113^. TSLP directly activates sensory neurons expressing transient receptor potential ankyrin 1, thereby triggering itch signaling^114^. Additionally, Th2 cytokines such as IL-4 directly stimulate afferent neurons *via* the IL-4 receptor *α* and JAK1^115^. TSLP and substance P amplify this process by activating the production of Th2 cytokines, creating a feedforward loop in which Th2 cytokines induce additional TSLP expression, perpetuating the itch^116^. Beyond peripheral sensitization mechanisms, dysregulation of central neural circuits also contributes to AD-associated pruritus. Peptidergic C fibers release neuropeptides such as substance P, exacerbating itch and neuroinflammation^117^. Moreover, non-peptidergic sensory neurons stimulated by IL-31 release brain natriuretic peptide, which transmits IL-31-mediated itch signals to the spinal cord, linking peripheral inflammation to central pruriceptive processing^118^^,^^119^.

Dysbiosis of the skin microbiome, particularly the abnormal colonization of *S. aureus*, also plays a significant role in AD pathogenesis^120^^,^^121^. Overgrowth of *S. aureus* exacerbates skin barrier dysfunction and inflammation through multiple mechanisms, such as the release of *δ*-toxin, which stimulates mast cell degranulation, and superantigens like enterotoxins, which activate T cells^122^^,^^123^. Recent studies have emphasized the role of vascular endothelial growth factor in AD by promoting angiogenesis^124^.

Specific transcription factors are integral to AD pathogenesis. Notably, NF-*κ*B not only enhances the transcription of Th2 cytokines but also induces cytokines like interferon-gamma (IFN-*γ*), modulating the delayed Th1-dominant allergic response in AD^125^, ^126^, ^127^. IFN-*γ* can upregulate cytokines such as CCL5, which recruits eosinophils and macrophages, and CCL8, which drives the recruitment of Th2 cells^128^, ^129^, ^130^, ^131^. Knockdown of *RELA* significantly reduces the levels of CCL5 and CCL8^107^. Hwang et al.^132^ demonstrated that upregulated IL-31 levels in AD mouse skin lesions are directly associated with increased infiltration of nuclear factor of activated T-cells 1 (NFAT1)^+^CD4^+^ T cells, with NFAT1 enhancing IL-31 expression. Additionally, transmembrane protein 232, significantly elevated in both MC903-induced AD mouse models and human AD lesions, exacerbates inflammation by activating NF-*κ*B and STAT3 pathways^133^. Guanine adenine thymine adenine sequence-binding protein 3 (GATA-3) and T-box expressed in T cells (T-bet) are master transcription factors for Th2 and Th1 cell differentiation and activation, respectively^134^. GATA-3 drives Th2 cells to produce IL-4, IL-5, and IL-13, while T-bet is essential for Th1 cells to produce IFN-*γ*^18^^,^^135^^,^^136^.

## 3 AD: Current therapeutic landscape

**Figure 4:**
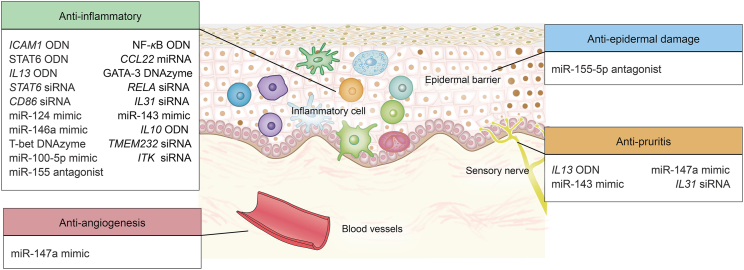
Pharmacological actions of antisense molecules as potential therapeutics for AD. ODN, antisense DNA; NF-*κ*B, nuclear factor kappa-B; STAT6, signal transducers and activators of transcription 6; T-bet, T-box expressed in T cells; GATA-3, guanine adenine thymine adenine sequence-binding protein 3.

Topical anti-inflammatory treatments remain the cornerstone of AD symptomatic control. TCS, with its multiple mechanisms—anti-inflammatory, immunosuppressive, vasoconstriction promotion, and so on, has long been the first-line treatment for AD^137^. As an alternative to TCS, topical calcineurin inhibitors (*e*.*g*., tacrolimus and pimecrolimus) exert their immunosuppressive effect by inhibiting the activation of T lymphocytes^138^. Phosphodiesterase-4 inhibitors have demonstrated clinical efficacy in AD patients by enhancing anti-inflammatory cytokine production (*e*.*g*., IL-10) while concurrently suppressing pro-inflammatory mediators (IL-4, IL-13, IL-22) and chemokine synthesis^139^^,^^140^. For mild-to-moderate AD, these therapies provide transient relief, but disease recurrence often necessitates prolonged use, leading to dose-dependent adverse effects such as skin atrophy, increased susceptibility to infections, metabolic disturbances, and malignancies^141^^,^^142^. In moderate-to-severe AD, topical agents exhibit minimal efficacy, necessitating escalation to systemic immunosuppressants like cyclosporine, a calcineurin inhibitor used systemically to treat AD^143^, methotrexate, or azathioprine, which use their cytotoxicity to inhibit cells, particularly T lymphocytes, and exert anti-inflammatory effects by inhibiting activation and adhesion molecules^144^^,^^145^. However, these approaches are plagued by non-specific immunosuppression, resulting in substantial off-target risks, such as renal toxicity, hepatosis, and leukopenia^19^. In recent years, the introduction of new targeted biologics and small-molecule drugs has brought new hope to AD patients. Dupilumab, a fully humanized monoclonal antibody that blocks the *α*-chain of IL-4 and IL-13 receptors, has a good curative effect in adults and children aged 6 years or older with moderate-to-severe AD and has been listed in many countries^15^. Nevertheless, in addition to being costly, it may suffer from conjunctivitis, herpes zoster, acne, or folliculitis^146^. Nemolizumab, which targets IL-31 receptor *α*, has demonstrated promising efficacy in alleviating itching and improving symptoms in AD patients aged 12 years and older^16^, but the efficacy of this biologic was only temporary, suggesting that IL-31 has no upstream role in the pathogenesis of AD^147^. JAK inhibitors such as abrocitinib, upadacitinib, and baricitinib have been approved for the systemic treatment of moderate-to-severe AD patients and have shown good therapeutic effects. However, this class of drug has been associated with hepatotoxicity, nephrotoxicity, and blood toxicity (including thrombocytopenia and lymphocytopenia), with black box warnings of increased mortality, malignancy, and major cardiovascular events^148^. Despite the availability of these new treatments, a substantial proportion of patients further face treatment resistance or partial responses^18^. Allergen immunotherapy (AIT), also known as desensitization therapy, refers to the gradual increase of patient exposure to specific allergen doses, enabling the immune system to progressively adapt and develop tolerance, ultimately achieving the aim of desensitization^149^. Several large randomized clinical trials^150^, ^151^, ^152^ have reported that this strategy can importantly improve AD severity and the quality of life of AD patients. However, AIT's adverse events increased, and no study addressed its potential long-term immunomodulatory effects. A critical limitation of current therapies is their inability to fully address the multi-pathogenic nature of AD, which encompasses dysregulated epidermal barrier function, Th2/Th1-driven inflammation, genetic and epigenetic changes, neuroimmune interactions, and other contributing factors^13^. This incomplete blockade of disease pathways perpetuates a “relapse upon discontinuation” cycle and further complicates long-term management. As a result, the development of safe and effective treatments that synergistically target upstream core disease drivers in AD remains a pressing medical priority, motivating researchers to investigate treatments such as oligonucleotide-based therapies (Fig. 4).

## 4 Antisense DNA and siRNA as therapeutic strategies for AD

ODNs and small interfering RNA (siRNA) have emerged as potent tools for the specific modulation of gene expression, gaining increasing acceptance for therapeutic applications (Table 1). While siRNAs demonstrate robust mRNA degradation capabilities in cellular cultures, their cell penetration lags behind ODNs and requires the use of cell-penetrating proteins^21^^,^^153^^,^^154^. The *in vivo* administration of siRNAs often suffers from poor specificity^155^^,^^156^ and immunogenicity, which can lead to severe toxicity^157^. To solve those problems, several chemical modification techniques for siRNAs, such as 2′-*O*-benzyl have been used^158^. ODNs are generally preferred due to their superior stability in both cellular and cell-free environments, as well as their simpler synthesis^159^^,^^160^. Nevertheless, the potential for ODNs to exhibit protein-binding activity must be carefully considered in their application^161^. Given their promise for AD therapy, substantial efforts have been directed toward developing these strategies. This section highlights key examples (Table 4^103^^,^^105^, ^106^, ^107^, ^108^^,^^132^^,^^133^^,^^162^, ^163^, ^164^, ^165^, ^166^, ^167^, ^168^, ^169^, ^170^, ^171^, ^172^, ^173^, ^174^, ^175^, ^176^, ^177^, ^178^, ^179^, ^180^, ^181^, ^182^, ^183^).

**Table 4:** Studies describing antisense molecules administered in AD therapy.

Antisense molecule	Target	Delivery vector	Administration	Model	Action	Adverse reaction	Ref.
ODN	*Icam1*^b^	Liposome	Intravenous	Mice	Inhibit lymphocyte trafficking and activation	Unreported	162
		Atelocollagen				Immunostimulation/Histological changes of liver and kidney	163
	*Il10*^b^	/	Iontophoresis	Mice	Inhibit IL-10	Unreported	173
	NF-*κ*B^b^	/	Topical application	Mice	Regulate Th1 and Th2 immune responses	Local immunosuppression causes infection and cancer	164
				Patient		Unreported	165
	STAT6^b^	HVJ–liposome	Subcutaneous/Intramuscular	Mice	Regulate Th2 immune responses and IgE production	Unreported	170
			Topical application	Patient			171
	*Il13*^b^	Cationic liposome	Topical application	Mice	Inhibit IL-13	No obvious side effects	174
siRNA	*Cd86*	Cream	Topical application	Mice	Inhibit T cell activation	Unreported	175
	*Stat6*	Cationic liposome	Subcutaneous	Mice	Regulate Th2 immune responses and IgE production	Th2/Th1 imbalances lead to increased risks of Th1-mediated disease progression	172
	*ITK*	/	Transfection	Cell	Inhibit T cell development, activation and function	Unreported	176
	*Rela*	Cell-penetrating peptide	Transfection/To-pical application	Cell/mice	Regulate Th1 and Th2 immune responses	Unreported	166, 167, 168, 169
	*Il31*	/	Transfection	Cell	Inhibit IL-31	Unreported	132
	*Tmem232*	/	Topical application	Mice	Activate the NF-*κ*B and STAT3 pathways	Unreported	133
miRNA	*Ccl22*	Recombinant *Salmonella typhimurium*	Transfection/Or-al	Cell/mice	Inhibit Th2 cell recruitment	No obvious side effects	177
	miR-146a	/	Transfection/To-pical application	Cell/mice	Inhibit IRAK1,TRAF6,CCL5,CCL8,CARD10,UBD	Unreported	105,106
	miR-155	/	Transfection	Cell	Inhibit SOCS1	Unreported	179
	miR-143^a^	/	Transfection	Cell	Inhibit IL-13R*α*1	Unreported	103
	miR-124^a^	/	Transfection	Cell	Inhibit RelA	Unreported	107
	miR-155-5p	/	Transfection/Su-bcutaneous	Cell/mice	Inhibit PKI	Unreported	180
	miR-147a^a^	Exosomes	Transfection	Cell	Inhibit VEGFA and MEF2A-TSLP axis	Unreported	108
	miR-100-5p^a^	OSA-CMCS hydrogel	Topical application	Mice	Reduce pyroptosis	Unreported	178
DNAzyme	*GATA3*	/	Transfection	Cell	Inhibit Th2 cell differentiation and activation	No off-targeted effects	181
			Topical application	Mice		Unreported	182
				Patient			183
	*TBX21*	/	Transfection/To-pical application	Cell/mice	Inhibit Th1 cell differentiation and activation	Unreported	182

NF-*κ*B, nuclear factor kappa-B; HVJ, hemagglutinating virus of JAPAN; CARD10, caspase recruitment domain-containing protein 10; IRAK1, IL-1 receptor–associated kinase 1; UBD, ubiquitin D; SOCS1, suppressor of cytokine signaling-1; PKI, protein kinase A inhibitor; MEF2A, myocyte enhancer factor 2A; TRAF6, tumor necrosis factor receptor-associated factor 6; TLR2, Toll-like receptor 4.

a Expression in AD patients is downregulated.

b The chemical modification of antisense molecule is phosphorothioate.

Hertl et al.^184^ demonstrated the efficacy of a phosphorothioate oligonucleotide (S-ODN) targeting the 3′ untranslated region of *ICAM1* mRNA. At concentrations ranging from 0.2 to 1.0 μmol/L, S-ODN abolished ICAM-1 expression in up to 75% of keratinocytes. Their work emphasized the importance of lipid-based delivery systems and ODN backbone structure in optimizing antisense efficacy and represented an early successful attempt to combine antisense ODN with lipid delivery systems for gene modulation. Further, atelocollagen-mediated delivery of S-ODN in inflamed mouse models showed over 50-fold greater efficacy compared to a single S-ODN infusion, suggesting enhanced systemic delivery and antisense efficacy^162^. Comparable results were observed in a murine AD model using S-ODN encapsulated with egg phosphatidylcholine and cholesterol^163^. However, the absence of pharmacokinetic data—such as half-life (*t*_1/2_), maximum blood concentration, and area under the curve—limits the ability to guide clinical dosing regimens, such as administration frequency and dose.

Nakamura et al.^164^ investigated the efficacy of an NF-*κ*B decoy ODN ointment in NC/Nga mice. The results demonstrated significant improvements in clinical skin scores and histology, with reduced mast cell migration and increased apoptotic cells. This ointment exhibited fewer prominent local or adverse effects compared to TCS. In a clinical trial with 10 severe adult AD patients, 6 out of 7 patients with facial inflammation responded favorably to the 2% NF-*κ*B decoy ointment. Although the trunk lesions showed no improvement, suggesting a possible site-dependent effect, the mechanism of which needs to be further explored (*e*.*g*., skin thickness). Further phase II trial revealed optimal efficacy at the medium dosage^165^. The NF-*κ*B target has also been validated in the siRNA strategy. Uchida et al. demonstrated effective *Rela* mRNA silencing in mouse dendritic cells using anti-*Rela* siRNA (siRela) complexed with the cell-penetrating peptide Tat, which improved nuclease resistance and transdermal delivery significantly^166^. Further optimization with Tat and AT1002 significantly improved ear thickness, skin severity, cytokine levels, and serum IgE in AD mice, enhancing siRNA stability and delivery efficiency greatly^167^. Another study employed siRela with a stearoyl-oligopeptide OK-102, demonstrating effective *Rela* knockdown without immunotoxicity, highlighting its clinical potential^168^. Ibaraki et al.^169^ used siRela complexed with a peptide STR-CH2R4H2C and AT1002, showing increased cellular uptake, reduced TNF-*α* and IL-6 expression, and significant symptom relief in AD mice. Despite the availability of various cell-penetrating proteins, systematic comparisons of their stability, delivery efficiency, and toxicity are lacking, making it difficult to determine the optimal strategy and hindering the timely progress of clinical trials for AD.

In targeting STAT6, siRNA and ODN approaches exhibit distinct developmental trajectories. In the ODN paradigm, Yokozeki et al.^170^ reported that subcutaneous STAT6 decoy ODN administration attenuated late-phase inflammatory responses in murine models through suppression of eosinophil/neutrophil infiltration and Th2 cell recruitment. This strategy demonstrated translational potential in a 7-patient clinical trial^171^ showing reduced refractory facial erythema and pruritus, though its broader applicability remains constrained by limited sample size and unverified long-term safety profiles. Hosoya's team^172^ screened three *Stat6* siRNA variants, identifying siRNA 3 as the optimal candidate through its superior suppression of STAT6 protein overexpression in dermal fibroblasts. Subcutaneous injection of siRNA 3 into the auricle of mice resulted in a marked reduction in contact hypersensitivity to trinitrochlorobenzene, dinitrofluorobenzene, and oxazolone, with histopathologic analysis confirming reduced dermal cell infiltration and edema. In summary, despite promising therapeutic prospects demonstrated by animal experiments, safety evaluations, and quantitative data on *t*_1/2_ and delivery efficiency of *Stat6* siRNA 3 remain incomplete. As for clinical studies, *Stat6* siRNA has yet to commence, and decoy ODN is still confined to the phase of localized application.

ODN therapy has also been studied for other AD-related targets. Arima et al.^185^ used an ODN for mouse *Il1*0 mRNA (AS6), which significantly decreased the production of IL-10 in RAW264.7, a murine macrophage-like cell line. Sakamoto et al.^173^ demonstrated that topical AS6 application in NC/Nga mice decreased IL-10 and its mRNA levels, leading to lesion resolution. The aforementioned study utilized AS6 derived from murine sources, and its safety and efficacy necessitate further validation through human cellular or clinical trials. Additionally, there was research using the *Il13* ODN/cationic elastic liposome combination, and the IL-13 secretion from Th2 cells was greatly inhibited. Treating the ovalbumin-sensitized murine models of AD with this complex significantly reduced the IL-13 production (by as much as 70% of the control) in the suffering skin regions as well as the IL-4 and IL-5 levels, alongside decreased inflammatory cell infiltration and skin thickness^174^.

There have been significant efforts made to develop siRNA therapeutics for AD. Hwang et al.^132^ transfected NFAT1 siRNA into activated CD4^+^ T cells, and IL-31 expression levels were considerably decreased, suggesting the potential of NFAT1 siRNA to treat IL-31-mediated AD symptoms. A study has shown the therapeutic efficacy of cream-emulsified *Cd86* siRNA in NC/Nga mice, resulting in significant improvement of AD clinical signs, along with decreased serum levels of IL-4, IgE, and IgG^175^. von Bonin et al.^176^ have proved that siRNA-mediated inhibition of IL-2-inducible tyrosine kinase in primary human T cells effectively suppressed T cell receptor-induced lymphokine production. Topical application of transmembrane protein 232 siRNA significantly ameliorated MC903-induced AD-like lesions in mice, reducing AD severity scores, ear thickness, and inflammatory cell infiltration. Concurrently, the phosphorylation levels of RelA and STAT3 were significantly suppressed, and mRNA levels of AD-related cytokines (*e*.*g*., IL-1*β*, IL-4, IL-5, IL-6, IL-13, IL-31, and IL-33) and chemokines (*e*.*g*., CCL17, CCL22) were notably downregulated^133^.

## 5 Targeting key miRNAs in the pathogenesis of AD

Since miRNAs are very stable in tissues and biofluids^186^^,^^187^, they can be identified with great specificity and sensitivity using high-throughput sequencing technology. Despite the fact that several miRNAs have been associated with AD, only a small number of them have been functionally validated in AD models to confirm their potential as therapeutic targets. Next, we review several key miRNA studies that highlight potential therapeutic benefits for AD (Table 4).

### 5.1 miRNA mimic

Zeng et al.^103^ showed that forced expression of miR-143 mimic in normal human epidermal keratinocytes could reverse the IL-13-induced downregulation of FLG, loricrin, and involucrin. Yoon et al.^177^ designed a recombinant strain of *Salmonella typhimurium* that expressed CCL22 miRNA, which downregulated CCL22 expression in activated lymphocytes. When mice with AD-like lesions were treated with *Ccl2*2 miRNA, CCL22 and IL-4 levels were suppressed, and IFN-*γ* levels were induced. Yang et al.^107^ identified that miR-124 directly suppressed the expression of RelA, suggesting its potential to regulate IFN-*γ*-induced cytokine production. And transfection of miR-124 mimics into IFN-*γ*-induced human keratinocytes significantly downregulated the levels of IL-8, CCL5, and CCL8.

In a research^105^ using miR-146a mimics in TLR2-induced human keratinocytes, the expression of IL-8, CCL20, and TNF-*α* was considerably inhibited, further repressing the recruitment of neutrophils. Furthermore, even under homeostatic conditions, miR-146a still suppressed the expression of inflammatory mediators and weakened the TLR2 stimulation in nonstimulated keratinocytes. In MC903-induced AD mouse models, the expression of miR-146a was enhanced in wild-type mice, while miR-146a-deficient mice showed more obvious inflammation, characterized by increased dermal infiltrating cells and increased production of IFN-*γ*, CCL5, and CCL8^106^. This suggests the potential of miR-146a to alleviate chronic skin inflammation in AD.

One study employed miR-147a-overexpressing exosomes in human epidermal keratinocyte (HaCaT) cells and human umbilical vein endothelial cells, which notably weakened inflammatory responses and cell apoptosis in HaCaT cells, and inhibited angiogenesis in human umbilical vein endothelial cells. In the mouse models of AD, miR-147a successfully inhibits pathological angiogenesis and inflammatory damage^108^. A study^178^ has evaluated the efficacy of miR-100-5p, which is highly expressed in extracellular vehicles (EVs) extracted from the mucus of *Pinctada fucata martensii*, for the treatment of AD. The results demonstrated that it had significant anti-inflammatory effects on HaCaT cells. Furthermore, *in vivo* experiments confirmed its ability to suppress inflammation and pyroptosis. Future research should focus on assessing the immunogenicity and other potential safety risks of these EVs as foreign substances in humans.

### 5.2 miRNA antagonist

In one study, transfection of miR-155 antagonists into cultured CD4^+^ T cells significantly reduced miR-155, Th17 cell differentiation, and IL17 expression, while markedly increasing SOCS1 expression^179^. This highlights the potential of miR-155 antagonists to modulate Th17-mediated immune response in AD. Wang et al.^180^ transfected miR-155-5p antagonists into HaCaT cells, protein kinase inhibitor, and TJ proteins (occludin, Zo-1, claudin) expression rose, whereas TSLP and IL-33 levels showed a marked decline. In the AD mouse models administered with miR-155-5p antagonists, molecular changes similar to the cell experiments were seen. Decreased Th2 cytokines release, inflammatory cell infiltration, and epidermal thickening were observed.

## 6 DNAzymes: Targeting transcription factors in AD

Since their first demonstration of *in vivo* activity in 1999^188^, 10–23 Dzs have shown significant therapeutic potential in preclinical studies across various animal models, positioning them as promising candidates for clinical applications^181^^,^^189^^,^^190^. Their catalytic activity, which is independent of intracellular enzymes, allows for flexible design and broad applicability^191^. Up to now, Dzs against chronic inflammatory illnesses are under clinical investigation, with two Phase II trials being successfully conducted (NCT01743768 and NCT02129439). Dz hgd40, in particular, has emerged as a strong candidate for Phase III trials^192^. As more clinical data become available, Dzs may prove to be a safe and effective treatment. This section highlights the progress of Dzs in AD therapy, focusing on their ability to target key transcription factors (Table 4).

The GATA-3-specific Dz hgd40, with 100% sequence identity between mice and humans. *In vitro*, hgd40 reduced GATA-3 protein and IL-13 production in human Th2-polarized CD4^+^ T cells compared to control Dzs^181^. Similarly, the human T-bet-specific Dz htd70 decreased T-bet and IFN-*γ* levels in human Th1-polarized CD4^+^ T cells^182^. *In vivo*, hgd40 mitigated oxazolone-induced skin swelling in AD mouse models, consistent with its efficacy in allergic asthma models, underscoring its broad therapeutic potential across target organs^182^^,^^193^. To enhance delivery, Schmidts et al.^194^^,^^195^ formulated hgd40 in a water-in-oil-in-water emulsion, improving penetration and protecting Dzs. A phase IIa clinical trial evaluated this formulation in 25 patients with AD (NCT02079688), though results remain unpublished^183^. For T-bet, the mouse-specific Dz mtd32 reduced skin swelling in both oxazolone-induced (mixed Th1/Th2 inflammation) and ovalbumin-induced (Th1-dominant inflammation) models^182^.

## 7 Challenges of using antisense therapeutic agents to treat AD

The above studies have shown that antisense molecules hold potential for treating AD; however, before putting them into clinical practice, several critical challenges require resolution, particularly in ensuring effective and safe delivery, prolonged target-specific action, and favorable pharmacokinetics *in vivo*. These issues are outlined below.

### 7.1 Inefficient delivery—instability, nucleases, and permeation

Although good effects of antisense molecules on gene regulation have been demonstrated, several major concerns remain*—*the instability, susceptibility to nuclease degradation, and low permeation efficiency. Recent efforts to optimize their efficiency have focused on chemical modifications and advanced delivery vectors (Table 4).

**Chemical Modifications.** To enhance stability and resistance to nuclease degradation, the backbone of antisense molecules has been modified with various chemical groups, such as phosphorothioate (PS), 2′-*O*-methyl, 2′-*O*-methoxymethyl (2′-MOE), 2′-fluoro, or phosphorodiamidate morphino (PMO)^196^. And currently approved antisense drugs are dominated by 2′-MOE (*e*.*g*., mipomersen^26^, nusinersen^27^, inotersen^29^, volanesoren^31^ and tofersen^34^) and PMO (*e*.*g*., eteplirsen^28^, golodirsen^30^, viltolarsen^32^ and casimersen^33^) modifications. In antisense therapeutics for AD, the most widely studied backbone variant is the PS. It not only protects the antisense molecules from degradation but also increases their hydrophobicity through sulfur atoms and further enhances their interaction with plasma proteins, making them have good pharmacokinetic properties^197^. Nevertheless, this modification is also linked to a number of unfavorable side effects^198^. In addition, further studies are needed to explore the role of other oligonucleotide modifications in the treatment of AD, such as second-generation chemical modifications, by modifying a ribose sugar based on the PS backbone, which have enhanced nuclease resistance and binding affinity, reduced toxicity, and immunogenicity. Third-generation chemical modifications that enhance target binding, such as nucleobase modifications (methylation on 5′ cytosines), 2′-MOE, and constrained ethyl groups^199^. Moreover, there has been a development of nucleic acids referred to as bicyclic and locked nucleic acids, which exhibit remarkable stability in the biological fluids and much-enhanced hybridization capabilities to targets^45^. However, the majority of modifications may also lead to changed toxicological profiles, necessitating a thorough examination of the adverse effects, such as the use of pharmacologically exaggerated doses to assess in rats^200^.

**Delivery Vectors.** Through chemical modifications, the stability of antisense molecules was greatly improved, but the permeation efficiency from the skin to the target cells was still unsatisfactory. As large, hydrophilic molecules, antisense molecules struggle to penetrate the skin barriers, including the SC and TJs^201^, ^202^, ^203^, ^204^. Increasing dosages is not a viable solution due to the risk of off-target effects. Physical enhancement techniques like electroporation, sonophoresis, and iontophoresis can promote transdermal delivery; nonetheless, less invasive and simpler topical administration strategies are needed since these procedures are intrusive and subject the skin to injury^166^. Nanodelivery systems, which include lipids, polymers, peptides, nucleotides, and inorganics, are popular for treating AD with antisense molecules^197^^,^^205^. The cationic elastic liposome has been shown to improve delivery as it is transformable enough to cross the epidermal barrier^202^. When applied topically with ODNs, its positive charge helps reduce systemic toxicity by restricting the complex to the afflicted region and facilitates easier cell membrane penetration compared to neutral or negatively charged particles^206^^,^^207^. It has already been reported that the atelocollagen/siRNA combination is very stable and effective in cell transduction^208^. Ritprajak et al.^175^ designed an anti-stimulatory cream-emulsified siRNA for infants, and it turned out to be more effective than the atelocollagen strategy. Lee et al.^209^ created a poly lactic-*co*-glycolic acid nanoparticle-based siRNA delivery approach with little cytotoxicity and efficient cellular uptake. Furthermore, they combined the nanoformulation with an ablative laser and showed a significant improvement in epidermis delivery. Hamasaki et al.^210^ identified new RNA interference (RNAi) drugs known as nkRNA1 and PnkRNA1, which have no immunogenicity and are more stable against nucleases than classical siRNA. In combination with stearoyl-oligopeptide OK-102, these new RNAi drugs have shown improved cellular uptake and endosomal escape in AD mouse models. Another study utilized oxidized sodium alginate-carboxymethyl chitosan hydrogel as a delivery vector to administer EVs in AD mice. This hydrogel exhibited several desirable properties, including stability, self-healing capability, excellent biocompatibility, and sustained release of EVs at the wound site. Animal experimental results further indicated that the hydrogel-EVs combination significantly alleviated inflammation in mice^178^. All in all, for AD's local antisense administration, the skin barrier penetration is largely enhanced with nanodelivery systems, but the target binding affinity still needs to be further improved.

### 7.2 Unsafe delivery—toxicity, target engagement, and immunogenicity

**Toxicity.** The administration methods of antisense drugs can significantly affect distribution and toxicity, which remains a key issue in their clinical applications. Common routes of administration include subcutaneous, intraperitoneal, intravenous, direct-to-site administration, etc^211^. When administered intravenously, systemic toxicity may occur. For instance, S-ODNs can stimulate the immune system and cause histological alterations in the kidney and liver^212^, ^213^, ^214^, while lipoplexes can cause the generation of pro-inflammatory cytokines^215^^,^^216^. Other systemic symptoms include flu-like symptoms, thrombocytopenia, morphologic changes (*e*.*g*., columnar, shrubby, and sessile microglia), and so on^217^^,^^218^. But unlike lipid-based delivery methods, polymer-based delivery systems, such as the ones employed with cyclodextrin polymer-based nanoparticle formulation containing siRNA and atelocollagen, do not seem to have the same immunostimulatory effects^219^^,^^220^. Owing to skin accessibility, the local application can effectively treat skin conditions without the liver's first-pass metabolism. While it minimizes systemic side effects, it may still cause infection and cancer due to local immunosuppression, and local injection site reactions (*e*.*g*., itching)^164^^,^^221^. Last but not least, it may be necessary to consider certain clinical side effects associated with the function of regulated genes. For example, a disruption in Th1/Th2 imbalance from STAT6 inhibition may predispose patients to Th1-mediated diseases^172^. Therefore, it is important to conduct a comprehensive assessment of the dysregulated network based on the individual condition of patients, rather than focusing on just one pathway. Overall, although two completed clinical trials reported no safety concerns^165^^,^^171^, the safety profile of other antisense strategies in AD is mostly unclear, with one Phase IIa trial (NCT02079688) yet not been reported (Table 5).

**Table 5:** Current AD clinical trials using antisense molecules.

Target	Dose	Primary end-point	Status	Details	Duration of exposure	Efficacy	Adverse reaction
NF-*κ*B	Qd/bid	/	Phase I completed	t.a. single-dose efficacy study/10 patients	1 month	Effective in facial inflammation, no relief from skin lesions on the trunk	Unreported
	/	Skin symptom scores	Phase II completed	t.a. multiple-dose-escalation efficacy/safety study	/	Improved symptom scores were observed in the moderate-dose group	Undiscovered
STAT6	Bid	EASI	Phase I completed	t.a. investigator-initiated, open-label pilot study/7 patients	4 weeks	Skin lesions and pruritus were clearly improved	Unreported
*GATA3*	10 mg bid	SCORAD	Phase IIa completed	t.a. single-center, randomized, vehicle-controlled, double-blinded efficacy/safety study/25 patients	15 days	Unreported	Unreported

NF-*κ*B, nuclear factor kappa-B; t.a., topical application; STAT6, signal transducers and activators of transcription 6; EASI, eczema area and severity index; SCORAD, scoring atopic dermatitis.

**Target engagement.** Off-target effects of antisense drugs include three main aspects: non-target organ accumulation and unintended RNA or protein binding^222^^,^^223^. One example is the finding that small hairpin RNAs produced by U6 RNA polymerase III may overwhelm the endogenous miRNA pathway and result in significant toxicity *in vivo*^224^. Enhancing target engagement at therapeutic doses is crucial for improving the efficacy and safety of antisense therapy. One efficient strategy is to conjugate antisense drugs with molecules (*e*.*g*., carbohydrates, antibodies, or aptamers) that bind specific cell membrane proteins^225^. For example, *N*-acetylgalactosamine coupled modification, which has high affinity with asialal glycoprotein receptor on hepatocytes, is one of the most widely used antisense drug delivery systems today, as seen in recently marketed drugs like givosiran^36^, lumasiran^37^, inclisiran^38^, vutrisiran^39^, and nedosiran^40^ (Table 1). Using the inherent specificity of antibodies to cell surface receptors, Sela's team conjugates ODN targeting the microtubule-associated protein tau with blood–brain barrier penetrating antibodies acting as a brainshuttle, showing good *in vitro* activity and *in vivo* pharmacokinetic behavior. It opens up new avenues for the treatment of tau-related neurodegenerative diseases such as Alzheimer's disease^226^. AD is often accompanied by increased antibody release, which makes it easy to employ antisense drugs that are conjugated with specific antibodies to enhance target binding. Selective organ-targeted lipid nanoparticles (LNPs), such as targeting the lung, spleen, and liver, have been successfully investigated^227^. Additionally, drugs conjugated with LNPs have been approved, like patisiran^41^ (Table 1). Although LNP delivery systems have been widely studied in preclinical studies of AD, their cellular targeting may need further investigation for treating AD. The latest study constructed an RNA 3D structure prediction model named RhoFold + combined with deep learning, which can accurately and efficiently predict the tertiary and secondary structure of RNA compared with other algorithms, and may help screen highly specific antisense sequences to avoid the risk of competition with RNA-binding proteins (such as splicing factors) or non-targeted RNAs^228^.

**Immunogenicity.** Innate immune response activation has been a crucial issue for the safety of antisense therapeutics. Antisense molecules introduced into cells are perceived as undesirable gene activity, which in turn stimulates the TLRs (TLR3, TLR7, and TLR8, etc.), RNA-dependent protein kinase, and so on^229^, ^230^, ^231^, ^232^, ^233^, ^234^, followed by the activation of inflammatory cytokines, the IFN-*γ* reactions, and the alternative pathway of the complement system. Symptoms from inflammatory cytokine release can be managed with supportive therapies or extended infusion periods^235^, while complement activation is transient and resolves within 24 h without significant adverse effects^219^. The innate immune system's activation depends on the length and sequence of antisense molecules. For example, synthetic siRNAs exceeding 30 bases with 5′ triphosphates demonstrate enhanced activation potentials^236^, ^237^, ^238^. Additionally, endosomal TLRs may be activated by “danger” sequences, such as GU-rich sequences, 5′GUCCUUCAA3′ and 5′UGUGU3′^239^. Since the expression cassette-derived RNAi effectors are transcribed in the nucleus, they will not cross the endosomal compartment to activate TLRs^240^, but may still stimulate immunity *via* unmethylated CpG islands within the expression cassettes^241^. While immunogenicity was a significant issue with the first-generation siRNA drugs^230^, substantial 2′-*O*-methyl base alterations in subsequent RNAi triggers have greatly solved this problem^242^. For ODNs, polyethylene glycol (PEG) modification of their vectors has reduced immunogenicity in some diseases^211^^,^^243^. Although antisense drugs exhibit generally low and clinically manageable immunogenicity, comprehensive monitoring remains essential for this novel therapeutic class. Given the diverse administration routes of these drugs, special consideration should be given to detecting anti-drug antibody subtypes in different samples (such as serum, cerebrospinal fluid, and tissue fluid) and their dynamic changes. To enable more holistic monitoring, samples should be collected and stored in the early stages of use, and immunogenicity should be investigated based on emerging clinical presentation and exposure data.

### 7.3 Pharmacokinetics

The pharmacokinetics of systemically administered oligonucleotides have been extensively studied, revealing key insights into their distribution, metabolism, and excretion. Using stem-loop quantitative real-time polymerase chain reaction, it was found that siRNA was significantly distributed in 7 target organs at 0.5 and 2 h post-intravenous injection. The liver exhibited the highest siRNA accumulation, followed by the spleen and kidney, while significantly lower levels were detected in the lung and heart. Notably, the duodenum and brain showed barely detectable amounts^244^. This organ-specific distribution aligns with previous observations of preferential renal uptake of PS-modified oligonucleotides. It has been demonstrated that the kidney plays a major role in the excretion of oligonucleotides, with the liver being the primary site of degradation^245^, ^246^, ^247^, ^248^. While these characteristics position the kidney as a potential therapeutic target for antisense therapies, they also raise concerns about nephrotoxicity risk, which calls for periodic monitoring^249^. Due to their relatively small size and negative charge, antisense drugs weakly bind to plasma proteins and are rapidly excreted by the kidneys, which results in poor pharmacokinetics and raises questions about their future use in AD^197^. Various carriers and modifications have been developed to enhance the size of antisense drugs, thus preventing glomerular filtration, improving their *t*_1/2_, and exhibiting good gene regulation at therapeutic doses. ALN-VSP employs LNPs to encapsulate siRNA, achieving the circulatory integrity for most siRNA and reaching the plateau level in 6–7 h^250^. Classical PEG modification prolongs the *t*_1/2_ by forming a peripheral hydration layer that prevents the clearance of the mononuclear macrophage system. The pharmacokinetics of Willebrand factor-targeted oligonucleotides conjugated to 40 kDa PEG were tested in monkeys with a half-life of 67 h after intravenous administration and a subcutaneous bioavailability of 98%. In blood samples taken 300 h later, the complex still showed 90% activity to inhibit Willebrand factor^251^. A novel approach utilizing the long cycle properties of human serum albumin, oligodeoxynucleotides targeting factor IXa were conjugated with the cysteine site of albumin, and the constructs showed high stability in serum-containing buffers and completely blocked factor IXa for 2 h^252^. At present, though many targets in AD have been validated in the preclinical trials, it is urgent to study the pharmacokinetics, safety profiles, and toxicology of antisense drugs in the treatment of AD, to enter clinical trials as soon as possible. Additionally, virtual clinical trial platforms could simulate the pharmacokinetics of various drug delivery regimens, speeding up the identification of optimal antisense strategies for treating AD^253^.

## 8 Current considerations and future perspectives

The current development of antisense therapies often focuses on isolated pathways; however, the complexity of AD necessitates a combinatorial targeting approach. Emerging technologies, such as integrating antisense molecules with CRISPR-based gene-modulating tools like CRISPR-Cas9, may offer a promising avenue to simultaneously regulate RNA expression and repair mutated genes, and mixed-mode strategies that combine ODN with siRNA may significantly broaden the therapeutic scopes of AD^254^^,^^255^. Furthermore, combining antisense therapies with skin microbiome regulators may provide a dual-dimensional approach to prevent disease recurrence by simultaneously targeting immune and microecological pathways. Except for miRNA, circular RNAs in ncRNAs exhibit functionality as miRNA sponges, translation templates, and transcriptional regulation, positioning them as high-value therapeutic targets and tools^256^. On the one hand, the ncRNA–miRNA–mRNA competing endogenous RNA network can be further improved to help identify hub nodes in AD-specific regulatory circuits for targeted antisense interventions, thereby reducing the risk associated with pan-targeted therapies^257^. On the other hand, engineered circular RNAs as miRNA sponges capable of sequestering multiple miRNAs have attenuated heart hypertrophy and preserved heart function in a mouse model of transverse aortic coarctation of heart disease, demonstrating their potential as a novel therapeutic tool that may be equally applicable in AD^258^.

Delivery Vectors is still being improved and optimized. For instance, the combination of Tat and AT1002 peptides with siRNA has shown promising efficacy in treating AD skin lacking SC^166^^,^^167^. To penetrate the SC efficiently, Kawai et al.^259^ developed a lyotropic liquid crystalline system encompassing Tat and AT1002, and this system considerably boosted intact skin permeability compared to other combinations. Realizing the full potential of antisense therapies requires advancements in endosomal escape technologies. One promising strategy involves conjuring cationic peptides with neutral PMOs, which in clinical trials of Duchenne muscular dystrophy was 18 times more active when given monthly than the FDA-approved non-cationic eteplirsen PMO given weekly^260^. But this strategy doesn't work with siRNA and ODN, due to their anionic phosphodiester and PS backbones, which will form ionic aggregates with cationic peptides. Another innovative strategy employs photosensitive nanocarriers that generate reactive oxygen species under near-infrared light irradiation, enabling precise spatiotemporal control of antisense molecule release^261^. However, repeated administration of nanocarriers often triggers antibody production, leading to rapid immune clearance^235^. To address this limitation, researchers have engineered nanocarriers coated with natural red blood cell membranes. This biomimetic approach not only evades immune detection but also enhances biocompatibility, reduces visceral organ accumulation, and minimizes toxic side effects^262^.

The heterogeneity of AD subtypes necessitates real-time monitoring of disease activity to guide antisense molecule selection and dosing. Stimuli-responsive hydrogel-based dynamic drug delivery platforms can be engineered to release antisense oligonucleotides in response to AD-specific biomarkers^263^^,^^264^, such as elevated IL-31 levels or pathological pH variations in lesional skin. Significant advancements have been made in leveraging microneedle delivery systems for AD therapy. Illustratively, researchers have successfully engineered novel nanoantibiotics by integrating EVs with hyaluronic acid-based microneedles, demonstrating efficacy in treating AD-associated bacterial infections^265^. Future research should prioritize the development of advanced platforms incorporating wearable microneedle sensors. These platforms can detect molecular levels indicative of disease activity in real time and transmit the signals into a closed-loop monitoring system, which immediately initiates microneedle-mediated precision drug delivery after decision analysis by artificial intelligence. These platforms have shown good applications in the management of chronic diseases such as diabetes^266^^,^^267^. By detecting cytokine fluctuations or RNA signatures, we will further explore this technology in AD antisense drug therapy to realize precision therapy. Similarly, the platforms can realize intelligent drug administration by monitoring the dynamic changes of organ-specific toxicity biomarkers. While the long-term therapeutic effects of antisense strategies offer advantages in addressing adherence issues, they also pose potential risks, particularly in vulnerable populations such as pregnant women. To mitigate these risks, high-affinity oligonucleotides that complement the siRNA guide chain have been developed to rapidly and efficiently reverse RNAi activity *in vivo*^268^.

Current clinical trials on the use of antisense molecules for the treatment of AD have shown encouraging results, yet further research is needed to fully realize their therapeutic potential. Future clinical trials should encompass larger and more diverse patient populations to ensure statistical significance and generalizability. Given that AD is a chronic condition, extending the duration of trials (beyond one year) is crucial for evaluating the long-term safety and efficacy. Subgroup analysis of patients can be performed based on age, disease severity, and genetic background, in which liquid biopsy techniques detecting RNA have been proven to successfully detect different stages of cancer, it can also be further developed in the future to classify AD patients into molecular subgroups, such as “barrier dominant” and “immunodominant”^269^^,^^270^. This stratification will help elucidate differential antisense treatment responses. Future trials should include active control groups, such as the current standard treatments, which will provide clearer insights into the relative efficacy and safety of antisense molecules.

The production of antisense therapies necessitates advanced synthesis technologies, exceptional precision, and stringent batch-to-batch consistency, all of which contribute to high manufacturing costs and play a critical role in the development and commercialization of antisense therapies. Consequently, a comprehensive evaluation of the economic feasibility of these therapies within patient populations is essential to ensure their widespread clinical adoption. Existing drugs have achieved significant results through the “pay for results” model. For instance, Harvard Pilgrim Healthcare's partnership with Amgen shows that tiered pricing based on clinical metrics like the six-dimensional evaluation system for rheumatoid arthritis patients can shift the payment standard from “drug usage” to “treatment effectiveness achieved”, significantly reducing costs. This mechanism also provides a replicable path for cost control of antisense drugs^271^. Microfluidic chip technology has shown breakthrough progress in the field of antisense drug production automation, and its core advantages are reflected in two aspects: First, its precision fluid control reduces synthesis cycles to 1/22 of conventional methods, enabling unprecedented production efficiency; Second, the platform facilitates on-demand drug development, as evidenced by its success in creating antisense candidates targeting SARS-CoV-2 genomic mRNA for COVID-19 treatment and exon 46 of *DMD* pre-mRNA for Duchenne muscular dystrophy. These advances suggest that the technique could also help produce and develop antisense candidates to treat AD^272^.

The therapeutic efficacy of antisense drugs in AD is intrinsically linked to patient-specific variables encompassing genetic and epigenetic polymorphisms, immune dysregulation patterns, and demographic determinants. Thus, personalized medicine should be a key focus in the development of antisense therapies. A precision medicine paradigm integrating multi-omics stratification (such as transcriptomics, epigenomics, and proteomics) with advanced computational techniques, including artificial intelligence-driven image analysis and natural language processing, enabling the elucidation of complex molecular networks and offering individual therapeutic targets in AD for antisense intervention, thereby enhancing therapeutic efficacy and minimizing the risk of adverse effects^273^. In addition, the use of animal models in dermatological research is limited not only for ethical considerations but also because of their inherent physiological disparities with human skin architecture. In contrast, advanced 3D bioprinting technology can print a variety of skin cells (such as melanocytes, endothelial cells, pericellular cells, and adipose cells) into a specific layer of the engineered skin constructs, achieving unprecedented biomimicry of native tissue and enhancement of regenerative function. Beyond replicating cutaneous physiology, this platform can harness precision medicine principles through patient-specific modeling: tumor microenvironment reconstruction using autologous cancer cells exemplifies its capacity for personalized drug discovery in oncology^274^. Such physiologically relevant models merit additional exploration for preclinical evaluation of antisense therapy in AD, enabling simultaneous assessment of target engagement efficiency, dermal penetration kinetics, and immunotoxicity profiles while maintaining patient-derived genetic signatures.

## 9 Conclusions

Contemporary therapeutic paradigms for AD predominantly adhere to classical single-target interventions, which provide transient symptom alleviation but frequently induce pharmacological dependence and tolerance. Moreover, the heterogeneity among patients and the spatio-temporal evolution of individual conditions make it difficult to treat all patients with only a few universal protocols, which poses a significant challenge for the treatment of AD. However, antisense molecule-based precision targeting strategies represent a novel therapeutic dimension for AD, with their multiple molecular intervention and disease modification capabilities offering potential breakthroughs beyond current therapeutic limitations. Unlike the easy production of antisense molecules, despite undergoing a complex screening process, aptamers were found not to bind to RNA targets in practical applications^275^^,^^276^. CRISPR-based gene-modulating tools targeting RNA have been developed recently, but they face challenges in the nuclear delivery of large Cas–gRNA complexes and may be more immunogenic than antisense molecules^277^, ^278^, ^279^. Furthermore, the prolonged therapeutic effects of antisense drugs align with AD's chronic nature, reducing treatment frequency burdens compared to short-acting JAK inhibitors. However, antisense therapy for AD remains in nascent stages compared to applications in other diseases. To accelerate clinical translation, the field must prioritize several critical pathways. Leverage multi-omics technologies to decode AD pathogenesis and identify core upstream therapeutic targets for individuals. Using microfluidic chip technology, RNA structure prediction models, and 3D bioprinting technology to develop and produce antisense drugs that can treat AD in a precise manner. Developing adaptive delivery platforms for antisense drugs to enable more efficient delivery and more precise control to suit AD's progression. Despite many preclinical studies showing the great potential of antisense drugs for AD, their clinical trials have stagnated in the era of the boom in small nucleic acid drugs. Global consortia should prioritize multicenter trials targeting NF-*κ*B, STAT6, and GATA-3, constructing AD-specific molecular subgroups through integrating multi-omics like transcriptomics and skin barrier lipidomics. By establishing AD-specific antisense molecule design guidelines, this approach may herald a new era of precision, durable AD therapeutics.

## Author contributions

Jiayi Xue: study concept and design, drafting of manuscript, visualization, and figure design. Zhirong Yao: study supervision, drafting of manuscript. All authors read and approved the final version to be published.

## Declaration of Generative AI and AI-assisted technologies in the writing process

During the preparation of this work, the authors used DeepSeek-R1 in order to improve the readability and language of the article. After using this tool, the authors reviewed and edited the content as needed and take full responsibility for the content of the publication.

## Conflicts of interest

The authors declare no conflicts of interest.

## References

[1] Wang C., Wei C.C., Wan L., Lin C.L., Tsai J.D. (2021). Association of exposure to hydrocarbon air pollution with the incidence of atopic dermatitis in children. Ital J Pediatr.

[2] Amagai M., Bolognia J.L., Schaffer J.V., Cerroni L. (2018).

[3] Chrostowska-Plak D., Reich A., Szepietowski J.C. (2013). Relationship between itch and psychological status of patients with atopic dermatitis. J Eur Acad Dermatol Venereol.

[4] Silverberg J.I., Gelfand J.M., Margolis D.J., Boguniewicz M., Boguniewicz M., Fonacier L. (2018). Patient burden and quality of life in atopic dermatitis in US adults: a population-based cross-sectional study. Ann Allergy Asthma Immunol.

[5] Silverberg J.I. (2019). Comorbidities and the impact of atopic dermatitis. Ann Allergy Asthma Immunol.

[6] Silverberg J.I., Gelfand J.M., Margolis D.J., Boguniewicz M., Fonacier L., Grayson M.H. (2018). Association of atopic dermatitis with allergic, autoimmune, and cardiovascular comorbidities in US adults. Ann Allergy Asthma Immunol.

[7] Paller A., Jaworski J.C., Simpson El, Boguniewicz M., Russell J.I., Block J.K. (2018). Major comorbidities of atopic dermatitis: beyond allergic disorders. Am J Clin Dermatol.

[8] Schmitt J., Langan S., Deckert S., Svensson A., von Kobyletzki L., Thomas K. (2013). Assessment of clinical signs of atopic dermatitis: a systematic review and recommendation. J Allergy Clin Immunol.

[9] Eyerich K., Novak N. (2013). Immunology of atopic eczema: overcoming the Th1/Th2 paradigm. Allergy.

[10] Paller A.S., Mina-Osorio P., Vekeman F., Boklage S., Mallya U.G., Ganguli S. (2022). Prevalence of type 2 inflammatory diseases in pediatric patients with atopic dermatitis: real-world evidence. J Am Acad Dermatol.

[11] Spergel J.M. (2010). Epidemiology of atopic dermatitis and atopic march in children. Immunol Allergy Clin.

[12] Drucker A.M., Wang A.R., Li W.Q., Sevetson E., Block J.K., Qureshi A.A. (2017). The burden of atopic dermatitis: summary of a report for the National Eczema Association. J Invest Dermatol.

[13] Langan S.M., Irvine A.D., Weidinger S. (2020). Atopic dermatitis. Lancet.

[14] Facheris P., Jeffery J., Del Duca E., Guttman-Yassky E. (2023). The translational revolution in atopic dermatitis: the paradigm shift from pathogenesis to treatment. Cell Mol Immunol.

[15] European Medicines Agency. Dupixent : EPAR - Product Information. Available from: https://www.ema.europa.eu/en/medicines/human/EPAR/dupixent. (Accessed 30 April 2025).

[16] Silverberg J.I., Wollenberg A., Reich A., Thaçi D., Legat F.J., Papp K.A. (2024). Nemolizumab with concomitant topical therapy in adolescents and adults with moderate-to-severe atopic dermatitis (ARCADIA 1 and ARCADIA 2): results from two replicate, double-blind, randomised controlled phase 3 trials. Lancet.

[17] Jinesh S., Radhakrishnan R. (2025). Pharmaceutical aspects of JAK inhibitors: a comparative review. Inflammopharmacology.

[18] Kychygina A., Cassagne M., Tauber M., Galiacy S., Paul C., Fournié P. (2022). Dupilumab-associated adverse events during treatment of allergic diseases. Clin Rev Allergy Immunol.

[19] Roekevisch E., Spuls P., Kuester D., Limpens J., Schmitt J. (2014). Efficacy and safety of systemic treatments for moderate-to-severe atopic dermatitis: a systematic review. J Allergy Clin Immunol.

[20] Miao Y., Fu C., Yu Z., Yu L., Y T., Wei M. (2024). Current status and trends in small nucleic acid drug development: leading the future. Acta Pharm Sin B.

[21] Potaczek D.P., Garn H., Unger S.D., Renz H. (2016). Antisense molecules: a new class of drugs. J Allergy Clin Immunol.

[22] Lu T.X., Rothenberg M.E. (2018). MicroRNA. J Allergy Clin Immunol.

[23] Lu T.X., Rothenberg M.E. (2013). Diagnostic, functional, and therapeutic roles of microRNA in allergic diseases. J Allergy Clin Immunol.

[24] Crooke S.T., Witztum Jl, Bennett C.F., Baker B.F. (2018). RNA-targeted therapeutics. Cell Metab.

[25] Perry C.M., Balfour J.A. (1999). Fomivirsen. Drugs.

[26] Hair P., Cameron F., McKeage K. (2013). Mipomersen sodium: first global approval. Drugs.

[27] Hoy S.M. (2017). Nusinersen: first global approval. Drugs.

[28] Syed Y.Y. (2016). Eteplirsen: first global approval. Drugs.

[29] Keam S.J. (2018). Inotersen: first global approval. Drugs.

[30] Heo Y.A. (2020). Golodirsen: first approval. Drugs.

[31] Paik J., Duggan S. (2019). Volanesorsen: first global approval. Drugs.

[32] Dhillon S. (2020). Viltolarsen: first approval. Drugs.

[33] Shirley M. (2021). Casimersen: first approval. Drugs.

[34] Blair H.A. (2023). Tofersen: first approval. Drugs.

[35] Nie T. (2024). Eplontersen: first approval. Drugs.

[36] Scott L.J. (2020). Givosiran: first approval. Drugs.

[37] Scott L.J., Keam S.J. (2021). Lumasiran: first approval. Drugs.

[38] Lamb Y.N. (2021). Inclisiran: first approval. Drugs.

[39] Keam S.J. (2022). Vutrisiran: first approval. Drugs.

[40] Syed Y.Y. (2023). Nedosiran: first approval. Drugs.

[41] Hoy S.M. (2018). Patisiran: first global approval. Drugs.

[42] Levin M.G., Tsao N.L., Singhal P., Liu C., Vy H.M.T., Paranjpe I. (2022). Genome-wide association and multi-trait analyses characterize the common genetic architecture of heart failure. Nat Commun.

[43] Naeem S., Zhang J., Zhang Y., Wang Y. (2024). Nucleic acid therapeutics: past, present, and future. Mol Ther Nucleic Acids.

[44] Yuen M., Lim S., Plesniak R., Tsuji K., Janssen H., Pojoga C. (2022). Efficacy and safety of bepirovirsen in chronic hepatitis B infection. N Engl J Med.

[45] Bennett C.F., Swayze E.E. (2010). RNA targeting therapeutics: molecular mechanisms of antisense oligonucleotides as a therapeutic platform. Annu Rev Pharmacol Toxicol.

[46] Shen X., Corey D.R. (2018). Chemistry, mechanism and clinical status of antisense oligonucleotides and duplex RNAs. Nucleic Acids Res.

[47] Crooke S.T., Liang X.H., Crooke R.M., Baker B.F., Geary R.S. (2021). Antisense drug discovery and development technology considered in a pharmacological context. Biochem Pharmacol.

[48] Kole R., Krainer A.R., Altman S. (2012). RNA therapeutics: beyond RNA interference and antisense oligonucleotides. Nat Rev Drug Discov.

[49] Dana H., Chalbatani G.M., Mahmoodzadeh H., Karimloo R., Rezaiean O., Moradzadeh A. (2017). Molecular mechanisms and biological functions of siRNA. Int J Biomed Sci.

[50] Bartel D.P. (2009). MicroRNAs: target recognition and regulatory functions. Cell.

[51] Rebane A., Akdis C.A. (2013). MicroRNAs: essential players in the regulation of inflammation. J Allergy Clin Immunol.

[52] Breaker R.R., Joyce G.F. (1994). Inventing and improving ribozyme function: rational design *versus* iterative selection methods. Trends Biotechnol.

[53] Bruno M.C., Gagliardi A., Mancuso A., Barone A., Tarsitano M., Cosco D. (2022). Oleic acid-based vesicular nanocarriers for topical delivery of the natural drug thymoquinone: improvement of anti-inflammatory activity. J Control Release.

[54] Aulton M.E. (2002).

[55] Nedoszytko B., Reszka E., Gutowska-Owsiak D., Trzeciak M., Lange M., Jarczak J. (2020). Genetic and epigenetic aspects of atopic dermatitis. Int J Mol Sci.

[56] Palmer C.N.A., Irvine A.D., Terron-Kwiatkowski A., Zhao Y., Liao H., Lee S.P. (2006). Common loss-of-function variants of the epidermal barrier protein filaggrin are a major predisposing factor for atopic dermatitis. Nat Genet.

[57] Moosbrugger-Martinz V., Leprince C., Méchin M.C., Simon M., Blunder S., Gruber R. (2022). Revisiting the roles of filaggrin in atopic dermatitis. Int J Mol Sci.

[58] Miajlovic H., Fallon P.G., Irvine A.D., Foster T.J. (2010). Effect of filaggrin breakdown products on growth of and protein expression by Staphylococcus aureus. J Allergy Clin Immunol.

[59] Thyssen J.P., Kezic S. (2014). Causes of epidermal filaggrin reduction and their role in the pathogenesis of atopic dermatitis. J Allergy Clin Immunol.

[60] Kezic S., Kemperman P.M.J.H., Koster E.S., de Jongh C.M., Thio H.B., Campbell L.E. (2008). Loss-of-function mutations in the filaggrin gene lead to reduced level of natural moisturizing factor in the stratum corneum. J Invest Dermatol.

[61] Brown S.J., McLean W.H.I. (2009). Eczema genetics: current state of knowledge and future goals. J Invest Dermatol.

[62] De Benedetto A., Rafaels N.M., McGirt L.Y., Ivanov A.I., Georas S.N., Cheadle C. (2011). Tight junction defects in patients with atopic dermatitis. J Allergy Clin Immunol.

[63] Budu-Aggrey A., Kilanowski A., Sobczyk M.K., Shringarpure S.S., Mitchell R., Reis K. (2023). European and multi-ancestry genome-wide association meta-analysis of atopic dermatitis highlights importance of systemic immune regulation. Nat Commun.

[64] Chi X., Jin W., Zhao X., Xie T., Shao J., Bai X. (2022). ROR*γ*t expression in mature TH17 cells safeguards their lineage specification by inhibiting conversion to TH2 cells. Sci Adv.

[65] Leonardi S., Cuppari C., Manti S., Filippelli M., Parisi G.F., Borgia F. (2015). Serum interleukin 17, interleukin 23, and interleukin 10 values in children with atopic eczema/dermatitis syndrome (AEDS): association with clinical severity and phenotype. Allergy Asthma Proc.

[66] Aktar M.K., Kido-Nakahara M., Furue M., Nakahara T. (2015). Mutual upregulation of endothelin-1 and IL-25 in atopic dermatitis. Allergy.

[67] Koga C., Kabashima K., Shiraishi N., Kobayashi M., Tokura Y. (2008). Possible pathogenic role of Th17 cells for atopic dermatitis. J Invest Dermatol.

[68] Lauffer F., Jargosch M., Baghin V., Krause L., Kempf W., Absmaier-Kijak M. (2020). IL-17C amplifies epithelial inflammation in human psoriasis and atopic eczema. J Eur Acad Dermatol Venereol.

[69] Aochi S., Tsuji K., Sakaguchi M., Huh N., Tsuda T., Yamanishi K. (2011). Markedly elevated serum levels of calcium-binding S100A8/A9 proteins in psoriatic arthritis are due to activated monocytes/macrophages. J Am Acad Dermatol.

[70] Ehrchen J.M., Sunderkötter C., Foell D., Vogl T., Roth J. (2009). The endogenous Toll-like receptor 4 agonist S100A8/S100A9 (calprotectin) as innate amplifier of infection, autoimmunity, and cancer. J Leukoc Biol.

[71] Halayko A.J., Ghavami S. (2009). S100A8/A9: a mediator of severe asthma pathogenesis and morbidity?. Can J Physiol Pharmacol.

[72] Silverberg N.B., Silverberg J.I. (2015). Inside out or outside in: does atopic dermatitis disrupt barrier function or does disruption of barrier function trigger atopic dermatitis?. Cutis.

[73] Schaeffer E.M., Yap G.S., Lewis C.M., Czar M.J., McVicar D.W., Cheever A.W. (2001). Mutation of Tec family kinases alters T helper cell differentiation. Nat Immunol.

[74] Perez-Villar J.J., Kanner S.B. (1999). Regulated association between the tyrosine kinase Emt/Itk/Tsk and phospholipase-C gamma 1 in human T lymphocytes. J Immunol.

[75] Larché M., Till S.J., Haselden B.M., North J., Barkans J., Corrigan C.J. (1998). Costimulation through CD86 is involved in airway antigen-presenting cell and T cell responses to allergen in atopic asthmatics. J Immunol.

[76] Tsuyuki S., Tsuyuki J., Einsle K., Kopf M., Coyle A.J. (1997). Costimulation through B7-2 (CD86) is required for the induction of a lung mucosal T helper cell 2 (TH2) immune response and altered airway responsiveness. J Exp Med.

[77] Furue M. (2020). Regulation of skin barrier function *via* competition between AHR axis *versus* IL-13/IL-4‒JAK‒STAT6/STAT3 axis: pathogenic and therapeutic implications in atopic dermatitis. J Clin Med.

[78] Huang L.H., Chung W.H., Wu P.C., Chen C.B. (2022). JAK‒STAT signaling pathway in the pathogenesis of atopic dermatitis: an updated review. Front Immunol.

[79] Nestle F.O., Di Meglio P., Qin J.Z., Nickoloff B.J. (2009). Skin immune sentinels in health and disease. Nat Rev Immunol.

[80] Klonowska J., Gleń J., Nowicki R.J., Trzeciak M. (2018). New cytokines in the pathogenesis of atopic dermatitis-new therapeutic targets. Int J Mol Sci.

[81] Xu M., Dong C. (2017). IL-25 in allergic inflammation. Immunol Rev.

[82] Imai T., Nagira M., Takagi S., Kakizaki M., Nishimura M., Wang J.B. (1999). Selective recruitment of CCR4-bearing Th2 cells toward antigen-presenting cells by the CC chemokines thymus and activation-regulated chemokine and macrophage-derived chemokine. Int Immunol.

[83] Katou F., Ohtani H., Nakayama T., Ono K., Matsushima K., Saaristo A. (2001). Macrophage-derived chemokine (MDC/CCL22) and CCR4 are involved in the formation of T lymphocyte-dendritic cell clusters in human inflamed skin and secondary lymphoid tissue. Am J Pathol.

[84] Nakazato J., Kishida M., Kuroiwa R., Fujiwara J., Shimoda M., Shinomiya N. (2008). Serum levels of Th2 chemokines, CCL17, CCL22, and CCL27, were the important markers of severity in infantile atopic dermatitis. Pediatr Allergy Immunol.

[85] Medzhitov R. (2001). Toll-like receptors and innate immunity. Nat Rev Immunol.

[86] Kumar H., Kawai T., Akira S. (2009). Pathogen recognition in the innate immune response. Biochem J.

[87] Sroka-Tomaszewska J., Trzeciak M. (2021). Molecular mechanisms of atopic dermatitis pathogenesis. Int J Mol Sci.

[88] Tsang M.S.M., Hou T., Chan B.C.L., Wong C.K. (2021). Immunological roles of NLR in allergic diseases and its underlying mechanisms. Int J Mol Sci.

[89] Ong P.Y., Ohtake T., Brandt C., Strickland I., Boguniewicz M., Ganz T. (2002). Endogenous antimicrobial peptides and skin infections in atopic dermatitis. N Engl J Med.

[90] Kaesler S., Volz T., Skabytska Y., Köberle M., Hein U., Chen K.M. (2014). Toll-like receptor 2 ligands promote chronic atopic dermatitis through IL-4-mediated suppression of IL-10. J Allergy Clin Immunol.

[91] Volz T., Skabytska Y., Guenova E., Chen K.M., Frick J.S., Kirschning C.J. (2014). Nonpathogenic bacteria alleviating atopic dermatitis inflammation induce IL-10-producing dendritic cells and regulatory Tr1 cells. J Invest Dermatol.

[92] Novak N., Simon D. (2011). Atopic dermatitis—from new pathophysiologic insights to individualized therapy. Allergy.

[93] van den Bogaard E.H., Elias P.M., Goleva E., Berdyshev E., Smits J.P.H., Danby S.G. (2023). Targeting skin barrier function in atopic dermatitis. J Allergy Clin Immunol Pract.

[94] Janssens M., van Smeden J., Gooris G.S., Bras W., Portale G., Caspers P.J. (2012). Increase in short-chain ceramides correlates with an altered lipid organization and decreased barrier function in atopic eczema patients. J Lipid Res.

[95] Hachem J.P., Wagberg F., Schmuth M., Crumrine D., Lissens W., Jayakumar A. (2006). Serine protease activity and residual LEKTI expression determine phenotype in Netherton syndrome. J Invest Dermatol.

[96] Igawa K. (2019). Future trends in the treatment of atopic dermatitis. Immunol Med.

[97] Yamamura K., Uruno T., Shiraishi A., Tanaka Y., Ushijima M., Nakahara T. (2017). The transcription factor EPAS1 links DOCK8 deficiency to atopic skin inflammation *via* IL-31 induction. Nat Commun.

[98] Williams H.C. (1995). Atopic eczema. BMJ.

[99] Mu Z., Zhang J. (2020). The role of genetics, the environment, and epigenetics in atopic dermatitis. Adv Exp Med Biol.

[100] Sonkoly E., Janson P., Majuri M.L., Savinko T., Fyhrquist N., Eidsmo L. (2010). MiR-155 is overexpressed in patients with atopic dermatitis and modulates T-cell proliferative responses by targeting cytotoxic T lymphocyte-associated antigen 4. J Allergy Clin Immunol.

[101] Yao R., Ma Y.L., Liang W., Li H.H., Ma Z.J., Yu X. (2012). MicroRNA-155 modulates Treg and Th17 cells differentiation and Th17 cell function by targeting SOCS1. PLoS One.

[102] Klingler C., Kniesel U., Bamforth S.D., Wolburg H., Engelhardt B., Risau W. (2000). Disruption of epithelial tight junctions is prevented by cyclic nucleotide-dependent protein kinase inhibitors. Histochem Cell Biol.

[103] Zeng Y.P., Nguyen G.H., Jin H.Z. (2016). MicroRNA-143 inhibits IL-13-induced dysregulation of the epidermal barrier-related proteins in skin keratinocytes *via* targeting to IL-13R*α*1. Mol Cell Biochem.

[104] Liang C., Liu Y., Xu H., Huang J., Shen Y., Chen F. (2021). Exosomes of human umbilical cord MSCs protect against hypoxia/reoxygenation-induced pyroptosis of cardiomyocytes *via* the miRNA-100-5p/FOXO3/NLRP3 pathway. Front Bioeng Biotechnol.

[105] Meisgen F., Xu Landén N., Wang A., Réthi B., Bouez C., Zuccolo M. (2014). MiR-146a negatively regulates TLR2-induced inflammatory responses in keratinocytes. J Invest Dermatol.

[106] Rebane A., Runnel T., Aab A., Maslovskaja J., Rückert B., Zimmermann M. (2014). MicroRNA-146a alleviates chronic skin inflammation in atopic dermatitis through suppression of innate immune responses in keratinocytes. J Allergy Clin Immunol.

[107] Yang Z., Zeng B., Wang C., Wang H., Huang P., Pan Y. (2017). MicroRNA-124 alleviates chronic skin inflammation in atopic eczema *via* suppressing innate immune responses in keratinocytes. Cell Immunol.

[108] Shi C., Pei S., Ding Y., Tao C., Zhu Y., Peng Y. (2023). Exosomes with overexpressed miR 147a suppress angiogenesis and infammatory injury in an experimental model of atopic dermatitis. Sci Rep.

[109] Wang J., Wu Z., Li D., Li N., Dindot S.V., Satterfield M.C. (2012). Nutrition, epigenetics, and metabolic syndrome. Antioxidants Redox Signal.

[110] Galler J.R., Rabinowitz D., Akbarian S., Lubin F. (2014). Progress in molecular biology and translational science.

[111] Herberth G., Bauer M., Gasch M., Hinz D., Röder S., Olek S. (2014). Maternal and cord blood miR-223 expression associates with prenatal tobacco smoke exposure and low regulatory T-cell numbers. J Allergy Clin Immunol.

[112] Martin M.J., Estravís M., García-Sánchez A., Dávila I., Isidoro-García M., Sanz C. (2020). Genetics and epigenetics of atopic dermatitis: an updated systematic review. Genes.

[113] Yosipovitch G., Berger T., Fassett M.S. (2020). Neuroimmune interactions in chronic itch of atopic dermatitis. J Eur Acad Dermatol Venereol.

[114] Wilson S.R., Thé L., Batia L.M., Beattie K., Katibah G.E., McClain S.P. (2013). The epithelial cell-derived atopic dermatitis cytokine TSLP activates neurons to induce itch. Cell.

[115] Oetjen L.K., Mack M.R., Feng J., Whelan T.M., Niu H., Guo C.J. (2017). Sensory neurons co-opt classical immune signaling pathways to mediate chronic itch. Cell.

[116] Mollanazar N.K., Smith P.K., Yosipovitch G. (2016). Mediators of chronic pruritus in atopic dermatitis: getting the itch out?. Clin Rev Allergy Immunol.

[117] Steinhoff M., Ständer S., Seeliger S., Ansel J.C., Schmelz M., Luger T. (2003). Modern aspects of cutaneous neurogenic inflammation. Arch Dermatol.

[118] Steinhoff M., Schmelz M., Szabó I.L., Oaklander A.L. (2018). Clinical presentation, management, and pathophysiology of neuropathic itch. Lancet Neurol.

[119] Mishra S.K., Hoon M.A. (2013). The cells and circuitry for itch responses in mice. Science.

[120] Kong H.H., Oh J., Deming C., Conlan S., Grice E.A., Beatson M.A. (2012). Temporal shifts in the skin microbiome associated with disease flares and treatment in children with atopic dermatitis. Genome Res.

[121] Leyden J.J., Marples R.R., Kligman A.M. (1974). *Staphylococcus aureus* in the lesions of atopic dermatitis. Br J Dermatol.

[122] Nakamura Y., Oscherwitz J., Cease K.B., Chan S.M., Muñoz-Planillo R., Hasegawa M. (2013). *Staphylococcus δ*-toxin induces allergic skin disease by activating mast cells. Nature.

[123] Macias E.S., Pereira F.A., Rietkerk W., Safai B. (2011). Superantigens in dermatology. J Am Acad Dermatol.

[124] Lee H.J., Hong Y.J., Kim M. (2021). Angiogenesis in chronic inflammatory skin disorders. Int J Mol Sci.

[125] Ko K.I., Merlet J.J., DerGarabedian B.P., Zhen H., Suzuki-Horiuchi Y., Hedberg M.L. (2022). NF-*κ*B perturbation reveals unique immunomodulatory functions in Prx1^+^ fibroblasts that promote development of atopic dermatitis. Sci Transl Med.

[126] Lin L., Spoor M.S., Gerth A.J., Brody S.L., Peng S.L. (2004). Modulation of Th1 activation and inflammation by the NF-kappaB repressor Foxj1. Science.

[127] Brunner P.M., Guttman-Yassky E., Leung D.Y. (2017). The immunology of atopic dermatitis and its reversibility with broad-spectrum and targeted therapies. J Allergy Clin Immunol.

[128] Fujita H., Shemer A., Suárez-Fariñas M., Johnson-Huang L.M., Tintle S., Cardinale I. (2011). Lesional dendritic cells in patients with chronic atopic dermatitis and psoriasis exhibit parallel ability to activate T-cell subsets. J Allergy Clin Immunol.

[129] Rebane A., Zimmermann M., Aab A., Baurecht H., Koreck A., Karelson M. (2012). Mechanisms of IFN-*γ*-induced apoptosis of human skin keratinocytes in patients with atopic dermatitis. J Allergy Clin Immunol.

[130] Appay V., Rowland-Jones S.L. (2001). RANTES: a versatile and controversial chemokine. Trends Immunol.

[131] Islam S.A., Chang D.S., Colvin R.A., Byrne M.H., McCully M.L., Moser B. (2011). Mouse CCL8, a CCR8 agonist, promotes atopic dermatitis by recruiting IL-5^+^ T_H_2 cells. Nat Immunol.

[132] Hwang J.S., Kim G.C., Park E., Kim J.E., Chae C.S., Hwang W. (2015). NFAT1 and JunB cooperatively regulate IL-31 gene expression in CD4^+^ T cells in health and disease. J Immunol.

[133] Han J., Cai X., Qin S., Zhang Z., Wu Y., Shi Y. (2023). TMEM232 promotes the inflammatory response in atopic dermatitis *via* the nuclear factor-*κ*B and signal transducer and activator of transcription 3 signalling pathways. Br J Dermatol.

[134] Ray A., Cohn L. (1999). Th2 cells and GATA-3 in asthma: new insights into the regulation of airway inflammation. J Clin Investig.

[135] Finotto S., De Sanctis G.T., Lehr H.A., Herz U., Buerke M., Schipp M. (2001). Treatment of allergic airway inflammation and hyperresponsiveness by antisense-induced local blockade of GATA-3 expression. J Exp Med.

[136] Klein-Hessling S., Jha M.K., Santner-Nanan B., Berberich-Siebelt F., Baumruker T., Schimpl A. (2003). Protein kinase A regulates GATA-3-dependent activation of IL-5 gene expression in Th2 cells. J Immunol.

[137] Ahluwalia A. (1998). Topical glucocorticoids and the skin—mechanisms of action: an update. Mediat Inflamm.

[138] Carr W.W. (2013). Topical calcineurin inhibitors for atopic dermatitis: review and treatment recommendations. Paediatr Drugs.

[139] Doherty A.M. (1999). Phosphodiesterase 4 inhibitors as novel anti-inflammatory agents. Curr Opin Chem Biol.

[140] Yang H., Wang J., Zhang X., Zhang Y., Qin Z.L., Wang H. (2019). Application of topical phosphodiesterase 4 inhibitors in mild to moderate atopic dermatitis: a systematic review and meta-analysis. JAMA Dermatol.

[141] Becker J.C., Houben R., Vetter C.S., Bröcker E.B. (2006). The carcinogenic potential of tacrolimus ointment beyond immune suppression: a hypothesis creating case report. BMC Cancer.

[142] Schäcke H., Döcke W.D., Asadullah K. (2002). Mechanisms involved in the side effects of glucocorticoids. Pharmacol Ther.

[143] Griffiths C.E., Katsambas A., Dijkmans B.A., Finlay A.Y., Ho V.C., Johnston A. (2006). Update on the use of ciclosporin in immune-mediated dermatoses. Br J Dermatol.

[144] Bateman E.A., Ardern-Jones M., Ogg G.S. (2007). Dose-related reduction in allergen-specific T cells associates with clinical response of atopic dermatitis to methotrexate. Br J Dermatol.

[145] Patel A.A., Swerlick R.A., McCall C.O. (2006). Azathioprine in dermatology: the past, the present, and the future. J Am Acad Dermatol.

[146] Reich K., Thyssen J.P., Blauvelt A., Eyerich K., Soong W., Rice Z.P. (2022). Efficacy and safety of abrocitinib *versus* dupilumab in adults with moderate-to-severe atopic dermatitis: a randomised, double-blind, multicentre phase 3 trial. Lancet.

[147] Ruzicka T., Mihara R. (2017). Anti-interleukin-31 receptor A antibody for atopic dermatitis. N Engl J Med.

[148] Yang X., Kambe N., Takimoto-Ito R., Kabashima K. (2021). Advances in the pathophysiology of atopic dermatitis revealed by novel therapeutics and clinical trials. Pharmacol Ther.

[149] Ca A.,M.A. (2011). Mechanisms of allergen-specific immunotherapy. J Allergy Clin Immunol.

[150] Novak N., Bieber T., Hoffmann M., Fölster-Holst R., Homey B., Werfel T. (2012). Efficacy and safety of subcutaneous allergen-specific immunotherapy with depigmented polymerized mite extract in atopic dermatitis. J Allergy Clin Immunol.

[151] Liu L., Chen J., Xu J., Yang Q., Gu C., Ni C. (2019). Sublingual immunotherapy of atopic dermatitis in mite-sensitized patients: a multi-centre, randomized, double-blind, placebo-controlled study. Artif Cells, Nanomed Biotechnol.

[152] Qin Y.E., Mao J.R., Sang Y.C., Li W.X. (2014). Clinical efficacy and compliance of sublingual immunotherapy with Dermatophagoides farinae drops in patients with atopic dermatitis. Int J Dermatol.

[153] Fu Z., Zhang X., Zhou X., Ur-Rehman U., Yu M., Liang H. (2021). *In vivo* self-assembled small RNAs as a new generation of RNAi therapeutics. Cell Res.

[154] Kumar L.D., Clarke A.R. (2007). Gene manipulation through the use of small interfering RNA (siRNA): from *in vitro* to *in vivo* applications. Adv Drug Deliv Rev.

[155] Jackson A.L., Burchard J., Schelter J., Chau B.N., Cleary M., Lim L. (2006). Widespread siRNA "off-target" transcript silencing mediated by seed region sequence complementarity. RNA.

[156] Birmingham A., Anderson E.M., Reynolds A., Ilsley-Tyree D., Leake D., Fedorov Y. (2006). 3′ UTR seed matches, but not overall identity, are associated with RNAi off-targets. Nat Methods.

[157] Judge A., MacLachlan I. (2008). Overcoming the innate immune response to small interfering RNA. Hum Gene Ther.

[158] Kenski D.M., Butora G., Willingham A.T., Cooper A.J., Fu W., Qi N. (2012). siRNA-optimized modifications for enhanced *in vivo* activity. Mol Ther Nucleic Acids.

[159] Mirabelli C.K., Bennett C.F., Anderson K., Crooke S.T. (1991). *In vitro* and *in vivo* pharmacologic activities of antisense oligonucleotides. Anti Cancer Drug Des.

[160] Crooke S.T., Lebleu B. (1993).

[161] Shen W., De Hoyos C.L., Migawa M.T., Vickers T.A., Sun H., Low A. (2019). Chemical modification of PS-ASO therapeutics reduces cellular protein-binding and improves the therapeutic index. Nat Biotechnol.

[162] Hanai K., Kurokawa T., Minakuchi Y., Maeda M., Nagahara S., Miyata T. (2004). Potential of atelocollagen-mediated systemic antisense therapeutics for inflammatory disease. Hum Gene Ther.

[163] Klimuk S.K., Semple S.C., Nahirney P.N., Mullen M.C., Bennett C.F., Scherrer P. (2000). Enhanced anti-inflammatory activity of a liposomal intercellular adhesion molecule-1 antisense oligodeoxynucleotide in an acute model of contact hypersensitivity. J Pharmacol Exp Therapeut.

[164] Nakamura H., Aoki M., Tamai K., Oishi M., Ogihara T., Kaneda Y. (2002). Prevention and regression of atopic dermatitis by ointment containing NF-*κ*B decoy oligodeoxynucleotides in NC/Nga atopic mouse model. Gene Ther.

[165] Tamai K.A. (2004). A nucleic acid-based medications for severe atopic dermatitis-clinical trial of NF-*κ*B decoy ointment. Igaku no Ayumi.

[166] Uchida T., Kanazawa T., Kawai M., Takashima Y., Okada H. (2011). Therapeutic effects on atopic dermatitis by anti-RelA short interfering RNA combined with functional peptides Tat and AT1002. J Pharmacol Exp Therapeut.

[167] Uchida T., Kanazawa T., Takashima Y., Okada H. (2011). Development of an efficient transdermal delivery system of small interfering RNA using functional peptides, Tat and AT-1002. Chem Pharm Bull (Tokyo).

[168] Kanazawa T., Hamasaki T., Endo T., Tamano K., Sogabe K., Seta Y. (2015). Functional peptide nanocarriers for delivery of novel anti-RelA RNA interference agents as a topical treatment of atopic dermatitis. Int J Pharm.

[169] Ibaraki H., Kanazawa T., Takashima Y., Okada H., Seta Y. (2016). Development of an innovative intradermal siRNA delivery system using a combination of a functional stearylated cytoplasm-responsive peptide and a tight junction-opening peptide. Molecules.

[170] Yokozeki H., Wu M.H., Sumi K., Awad S., Satoh T., Katayama I. (2004). *In vivo* transfection of a cis element 'decoy' against signal transducers and activators of transcription 6 (STAT6)-binding site ameliorates IgE-mediated late-phase reaction in an atopic dermatitis mouse model. Gene Ther.

[171] Igawa K., Satoh T., Yokozeki H. (2009). A therapeutic effect of STAT6 decoy oligodeoxynucleotide ointment in atopic dermatitis: a pilot study in adults. Br J Dermatol.

[172] Hosoya K., Satoh T., Yamamoto Y., Saeki K., Igawa K., Okano M. (2011). Gene silencing of STAT6 with siRNA ameliorates contact hypersensitivity and allergic rhinitis. Allergy.

[173] Sakamoto T., Miyazaki E., Aramaki Y., Arima H., Takahashi M., Kato Y. (2004). Improvement of dermatitis by iontophoretically delivered antisense oligonucleotides for interleukin-10 in NC/Nga mice. Gene Ther.

[174] Kim S.T., Lee K.M., Park H.J., Jin S.E., Ahn W.S., Kim C.K. (2009). Topical delivery of interleukin-13 antisense oligonucleotides with cationic elastic liposome for the treatment of atopic dermatitis. J Gene Med.

[175] Ritprajak P., Hashiguchi M., Azuma M. (2008). Topical application of cream-emulsified CD86 siRNA ameliorates allergic skin disease by targeting cutaneous dendritic cells. Mol Ther.

[176] von Bonin A., Rausch A., Mengel A., Hitchcock M., Krüger M., von Ahsen O. (2011). Inhibition of the IL-2-inducible tyrosine kinase (Itk) activity: a new concept for the therapy of inflammatory skin diseases. Exp Dermatol.

[177] Yoon W.S., Lee S.S., Chae Y.S., Park Y.K. (2011). Therapeutic effects of recombinant Salmonella typhimurium harboring CCL22 miRNA on atopic dermatitis-like skin in mice. Exp Mol Med.

[178] Wu Z., He L., Yan L., Tan B., Ma L., He G. (2024). Hydrogels treat atopic dermatitis by transporting marine-derived miR-100-5p-abundant extracellular vesicles. ACS Biomater Sci Eng.

[179] Ma L., Xue H.B., Wang F., Shu C.M., Zhang J.H. (2015). MicroRNA-155 may be involved in the pathogenesis of atopic dermatitis by modulating the differentiation and function of T helper type 17 (Th17) cells. Clin Exp Immunol.

[180] Wang X., Chen Y., Yuan W., Yao L., Wang S., Jia Z. (2019). MicroRNA-155-5p is a key regulator of allergic inflammation, modulating the epithelial barrier by targeting PKI. α. Cell Death Dis.

[181] Krug N., Hohlfeld J.M., Kirsten A.M., Kornmann O., Beeh K.M., Kappeler D. (2015). Allergen-induced asthmatic responses modified by a GATA3-specific DNAzyme. N Engl J Med.

[182] Purath U., Ibrahim R., Zeitvogel J., Renz H., Runkel F., Schmidts T. (2016). Efficacy of T-cell transcription factor-specific DNAzymes in murine skin inflammation models. J Allergy Clin Immunol.

[183] Yan J., Ran M., Shen X., Zhang H. (2023). Therapeutic DNAzymes: from structure design to clinical applications. Adv Mater.

[184] Hertl M., Neckers L.M., Katz S.I. (1995). Inhibition of interferon-gamma-induced intercellular adhesion molecule-1 expression on human keratinocytes by phosphorothioate antisense oligodeoxynucleotides is the consequence of antisense-specific and antisense-non-specific effects. J Invest Dermatol.

[185] Arima H., Takahashi M., Aramaki Y., Sakamoto T., Tsuchiya S. (1998). Specific inhibition of interleukin-10 production in murine macrophage-like cells by phosphorothioate antisense oligonucleotides. Antisense Nucleic Acid Drug Dev.

[186] Chen X., Ba Y., Ma L., Cai X., Yin Y., Wang K. (2008). Characterization of microRNAs in serum: a novel class of biomarkers for diagnosis of cancer and other diseases. Cell Res.

[187] Mitchell P.S., Parkin R.K., Kroh E.M., Fritz B.R., Wyman S.K., Pogosova-Agadjanyan E.L. (2008). Circulating microRNAs as stable blood-based markers for cancer detection. Proc Natl Acad Sci U S A.

[188] Santiago F.S., Lowe H.C., Kavurma M.M., Chesterman C.N., Baker A., Atkins D.G. (1999). New DNA enzyme targeting Egr-1 mRNA inhibits vascular smooth muscle proliferation and regrowth after injury. Nat Med.

[189] Ryoo S.R., Jang H., Kim K.S., Lee B., Kim K.B., Kim Y.K. (2012). Functional delivery of DNAzyme with iron oxide nanoparticles for hepatitis C virus gene knockdown. Biomaterials.

[190] Fahmy R.G., Dass C.R., Sun L.Q., Chesterman C.N., Khachigian L.M. (2003). Transcription factor Egr-1 supports FGF-dependent angiogenesis during neovascularization and tumor growth. Nat Med.

[191] Fokina A.A., Stetsenko D.A., François J.C. (2015). DNA enzymes as potential therapeutics: towards clinical application of 10-23 DNAzymes. Expet Opin Biol Ther.

[192] Sterna Biologicals. sterna biologicals raises further EUR 10.0 million (approx. USD 11.9 million) in series A - 2nd closing private placement. Available from: https://www.sterna-biologicals.com/company-news/32sterna-biologicals-raises-further-eur-10-0-million-approx-usd-119-million-in-series-a-2nd-closing-private-placement. (Accessed 7 January 2021).

[193] Sel S., Wegmann M., Dicke T., Henke W., Yildirim A.O., Renz H. (2008). Effective prevention and therapy of experimental allergic asthma using a GATA-3-specific DNAzyme. J Allergy Clin Immunol.

[194] Schmidts T., Dobler D., von den Hoff S., Schlupp P., Garn H., Runkel F. (2011). Protective effect of drug delivery systems against the enzymatic degradation of dermally applied DNAzyme. Int J Pharm.

[195] Schmidts T., Dobler D., Schlupp P., Nissing C., Garn H., Runkel F. (2010). Development of multiple W/O/W emulsions as dermal carrier system for oligonucleotides: effect of additives on emulsion stability. Int J Pharm.

[196] Miller C.M., Tanowitz M., Donner A.J., Prakash T.P., Swayze E.E., Harris E.N. (2018). Receptor-mediated uptake of phosphorothioate antisense oligonucleotides in different cell types of the liver. Nucleic Acid Therapeut.

[197] Johannes L., Lucchino M. (2018). Current challenges in delivery and cytosolic translocation of therapeutic RNAs. Nucleic Acid Therapeut.

[198] Winkler J., Stessl M., Amartey J., Noe C.R. (2010). Off-target effects related to the phosphorothioate modification of nucleic acids. ChemMedChem.

[199] Sheng L., Rigo F., Bennett C.F., Krainer A.R., Hua Y. (2020). Comparison of the efficacy of MOE and PMO modifications of systemic antisense oligonucleotides in a severe SMA mouse model. Nucleic Acids Res.

[200] Janas M.M., Schlegel M.K., Harbison C.E., Yilmaz V.O., Jiang Y., Parmar R. (2018). Selection of GalNAc-conjugated siRNAs with limited off-target-driven rat hepatotoxicity. Nat Commun.

[201] Proksch E., Brandner J.M., Jensen J.M. (2008). The skin: an indispensable barrier. Exp Dermatol.

[202] Murthy S.N., Sen A., Hui S.W. (2004). Surfactant-enhanced transdermal delivery by electroporation. J Control Release.

[203] Akhtar S. (1998). Antisense technology: selection and delivery of optimally acting antisense oligonucleotides. J Drug Target.

[204] Akhtar S., Kole R., Juliano R.L. (1991). Stability of antisense DNA oligodeoxynucleotide analogs in cellular extracts and sera. Life Sci.

[205] Ramasamy T., Ruttala H.B., Munusamy S., Chakraborty N., Kim J.O. (2022). Nano drug delivery systems for antisense oligonucleotides (ASO) therapeutics. J Control Release.

[206] Graf C., Gao Q., Schütz I., Noufele C., Ruan W., Posselt U. (2012). Surface functionalization of silica nanoparticles supports colloidal stability in physiological media and facilitates internalization in cells. Langmuir.

[207] Wraight C.J., White P.J. (2001). Antisense oligonucleotides in cutaneous therapy. Pharmacol Ther.

[208] Minakuchi Y., Takeshita F., Kosaka N., Sasaki H., Yamamoto Y., Kouno M. (2004). Atelocollagen-mediated synthetic small interfering RNA delivery for effective gene silencing *in vitro* and *in vivo. Nucleic Acids Res*.

[209] Lee W.R., Chou W.L., Lin Z.C., Sung C.T., Lin C.Y., Fang J.Y. (2022). Laser-assisted nanocarrier delivery to achieve cutaneous siRNA targeting for attenuating psoriasiform dermatitis. J Control Release.

[210] Hamasaki T., Suzuki H., Shirohzu H., Matsumoto T., D'Alessandro-Gabazza C.N., Gil-Bernabe P. (2012). Efficacy of a novel class of RNA interference therapeutic agents. PLoS One.

[211] Grabowska-Pyrzewicz W., Want A., Leszek J., Wojda U. (2021). Antisense oligonucleotides for Alzheimer's disease therapy: from the mRNA to miRNA paradigm. EBioMedicine.

[212] Levin A.A. (1999). A review of the issues in the pharmacokinetics and toxicology of phosphorothioate antisense oligonucleotides. Biochim Biophys Acta.

[213] Monteith D.K., Levin A.A. (1999). Synthetic oligonucleotides: the development of antisense therapeutics. Toxicol Pathol.

[214] Henry S.P., Templin M.V., Gillett N., Rojko J., Levin A.A. (1999). Correlation of toxicity and pharmacokinetic properties of a phosphorothioate oligonucleotide designed to inhibit ICAM-1. Toxicol Pathol.

[215] Tousignant J.D., Gates A.L., Ingram L.A., Johnson C.L., Nietupski J.B., Cheng S.H. (2000). Comprehensive analysis of the acute toxicities induced by systemic administration of cationic lipid:plasmid DNA complexes in mice. Hum Gene Ther.

[216] Loisel S., Le Gall C., Doucet L., Ferec C., Floch V. (2001). Contribution of plasmid DNA to hepatotoxicity after systemic administration of lipoplexes. Hum Gene Ther.

[217] Palumbo M.C., Gautam M., Sonneborn A., Kim K., Wilmarth P.A., Reddy A.P. (2023). MicroRNA137-loaded lipid nanoparticles regulate synaptic proteins in the prefrontal cortex. Mol Ther.

[218] Khetarpal S.A., Wang M., Khera A.V. (2019). Volanesorsen, familial chylomicronemia syndrome, and thrombocytopenia. N Engl J Med.

[219] Zuckerman J.E., Gritli I., Tolcher A., Heidel J.D., Lim D., Morgan R. (2014). Correlating animal and human phase Ia/Ib clinical data with CALAA-01, a targeted, polymer-based nanoparticle containing siRNA. Proc Natl Acad Sci U S A.

[220] Sano A., Maeda M., Nagahara S., Ochiya T., Honma K., Itoh H. (2003). Atelocollagen for protein and gene delivery. Adv Drug Deliv Rev.

[221] Thomas G.S., Cromwell W.C., Ali S., Chin W., Flaim J.D., Davidson M. (2013). Mipomersen, an apolipoprotein B synthesis inhibitor, reduces atherogenic lipoproteins in patients with severe hypercholesterolemia at high cardiovascular risk: a randomized, double-blind, placebo-controlled trial. J Am Coll Cardiol.

[222] Merkel O.M., Beyerle A., Beckmann B.M., Zheng M., Hartmann R.K., Stöger T. (2011). Polymer-related off-target effects in non-viral siRNA delivery. Biomaterials.

[223] Jackson A.L., Bartz S.R., Schelter J., Kobayashi S.V., Burchard J., Mao M. (2003). Expression profiling reveals off-target gene regulation by RNAi. Nat Biotechnol.

[224] Grimm D., Streetz K.L., Jopling C.L., Storm T.A., Pandey K., Davis C.R. (2006). Fatality in mice due to oversaturation of cellular microRNA/short hairpin RNA pathways. Nature.

[225] Dong Y., Siegwart D.J., Anderson D.G. (2019). Strategies, design, and chemistry in siRNA delivery systems. Adv Drug Deliv Rev.

[226] Sela T., Mansø M., Siegel M., Marban-Doran C., Ducret A., Niewöhner J. (2023). Diligent design enables antibody-ASO conjugates with optimal pharmacokinetic properties. Bioconjug Chem.

[227] Cheng Q., Wei T., Farbiak L., Johnson L.T., Dilliard S.A., Siegwart D.J. (2020). Selective organ targeting (SORT) nanoparticles for tissue-specific mRNA delivery and CRISPR-Cas gene editing. Nat Nanotechnol.

[228] Shen T., Hu Z., Sun S., Liu D., Wong F., Wang J. (2024). Accurate RNA 3D structure prediction using a language model-based deep learning approach. Nat Methods.

[229] Tanji H., Ohto U., Shibata T., Taoka M., Yamauchi Y., Isobe T. (2015). Toll-like receptor 8 senses degradation products of single-stranded RNA. Nat Struct Mol Biol.

[230] Kleinman M.E., Yamada K., Takeda A., Chandrasekaran V., Nozaki M., Baffi J.Z. (2008). Sequence- and target-independent angiogenesis suppression by siRNA *via* TLR3. Nature.

[231] Heil F., Hemmi H., Hochrein H., Ampenberger F., Kirschning C., Akira S. (2004). Species-specific recognition of single-stranded RNA *via* toll-like receptor 7 and 8. Science.

[232] Diebold S.S., Kaisho T., Hemmi H., Akira S., Reis e Sousa C. (2004). Innate antiviral responses by means of TLR7-mediated recognition of single-stranded RNA. Science.

[233] García M.A., Gil J., Ventoso I., Guerra S., Domingo E., Rivas C. (2006). Impact of protein kinase PKR in cell biology: from antiviral to antiproliferative action. Microbiol Mol Biol Rev.

[234] Hornung V., Ellegast J., Kim S., Brzózka K., Jung A., Kato H. (2006). 5′-Triphosphate RNA is the ligand for RIG-I. Science.

[235] Zuckerman J.E., Davis M.E. (2015). Clinical experiences with systemically administered siRNA-based therapeutics in cancer. Nat Rev Drug Discov.

[236] Caplen N.J., Parrish S., Imani F., Fire A., Morgan R.A. (2001). Specific inhibition of gene expression by small double-stranded RNAs in invertebrate and vertebrate systems. Proc Natl Acad Sci U S A.

[237] Kim D.H., Behlke M.A., Rose S.D., Chang M.S., Choi S., Rossi J.J. (2005). Synthetic dsRNA Dicer substrates enhance RNAi potency and efficacy. Nat Biotechnol.

[238] Robbins M.A., Li M., Leung I., Li H., Boyer D.V., Song Y. (2006). Stable expression of shRNAs in human CD34^+^ progenitor cells can avoid induction of interferon responses to siRNAs. in vitro. Nat Biotechnol.

[239] Judge A.D., Sood V., Shaw J.R., Fang D., McClintock K., MacLachlan I. (2005). Sequence-dependent stimulation of the mammalian innate immune response by synthetic siRNA. Nat Biotechnol.

[240] Maepa M.B., Ely A., Grayson W., Arbuthnot P. (2017). Sustained inhibition of HBV replication *in vivo* after systemic injection of AAVs encoding artificial antiviral primary microRNAs. Mol Ther Nucleic Acids.

[241] Yu E.P.K., Bennett M.R. (2014). Mitochondrial DNA damage and atherosclerosis. Trends Endocrinol Metabol.

[242] Robbins M., Judge A., Liang L., McClintock K., Yaworski E., MacLachlan I. (2007). 2′-*O*-Methyl-modified RNAs act as TLR7 antagonists. Mol Ther.

[243] Zalipsky S., Harris J.M., Harris J.M., Zalipsky S. (1997). Poly(ethylene glycol). chemistry and biological applications.

[244] Rappaport J., Hanss B., Kopp J.B., Copeland T.D., Bruggeman L.A., Coffman T.M. (1995). Transport of phosphorothioate oligonucleotides in kidney: implications for molecular therapy. Kidney Int.

[245] Goodchild J., Kim B., Zamecnik P.C. (1991). The clearance and degradation of oligodeoxynucleotides following intravenous injection into rabbits. Antisense Res Dev.

[246] Cossum P.A., Truong L., Owens S.R., Markham P.M., Shea J.P., Crooke S.T. (1994). Pharmacokinetics of a ^14^C-labeled phosphorothioate oligonucleotide, ISIS 2105, after intradermal administration to rats. J Pharmacol Exp Therapeut.

[247] Goodarzi G., Watabe M., Watabe K. (1992). Organ distribution and stability of phosphorothioated oligodeoxyribonucleotides in mice. Biopharm Drug Dispos.

[248] Agrawal S., Temsamani J., Tang J.Y. (1991). Pharmacokinetics, biodistribution, and stability of oligodeoxynucleotide phosphorothioates in mice. Proc Natl Acad Sci U S A.

[249] Sarmiento U.M., Perez J.R., Becker J.M., Narayanan R. (1994). *In vivo* toxicological effects of rel A antisense phosphorothioates in CD-1 mice. Antisense Res Dev.

[250] Tabernero J., Shapiro G.I., LoRusso P.M., Cervantes A., Schwartz G.K., Weiss G.J. (2013). First-in-humans trial of an RNA interference therapeutic targeting VEGF and KSP in cancer patients with liver involvement. Cancer Discov.

[251] Siller-Matula J.M., Merhi Y., Tanguay J.F., Duerschmied D., Wagner D.D., McGinness K.E. (2012). ARC15105 is a potent antagonist of von Willebrand factor mediated platelet activation and adhesion. Arterioscler Thromb Vasc Biol.

[252] Kuhlmann M., Hamming J.B.R., Voldum A., Tsakiridou G., Larsen M.T., Schmøkel J.S. (2017). An albumin-oligonucleotide assembly for potential combinatorial drug delivery and half-life extension applications. Mol Ther Nucleic Acids.

[253] Simulations Plus Investor Relations. Simulations plus releases GastroPlus® X, the next generation PBPK/PBBM modeling & simulation software. Available from: https://www.businesswire.com/news/home/20240515037814/en/Simulations-Plus-Releases-GastroPlus-X-The-Next-Generation-PBPKPBBM-Modeling-Simulation-Software. (Accessed 15 May 2024).

[254] Smits J.P.H., van den Brink N.J.M., Meesters L.D., Hamdaoui H., Niehues H., Jansen P.A.M. (2023). Investigations into the FLG null phenotype: showcasing the methodology for CRISPR/Cas9 editing of human keratinocytes. J Invest Dermatol.

[255] Lennox K.A., Behlke M.A. (2016). Cellular localization of long non-coding RNAs affects silencing by RNAi more than by antisense oligonucleotides. Nucleic Acids Res.

[256] Liu R., Zhang L., Zhao X., Liu J., Chang W., Zhou L. (2022). circRNA: regulatory factors and potential therapeutic targets in inflammatory dermatoses. J Cell Mol Med.

[257] Wang X., Bao K., Wu P., Yu X., Wang C., Ji L. (2018). Integrative analysis of lncRNAs, miRNAs, and mRNA-associated ceRNA network in an atopic dermatitis recurrence model. Int J Mol Sci.

[258] Lavenniah A., Luu T.D.A., Li Y.P., Lim T.B., Jiang J., Ackers-Johnson M. (2020). Engineered circular RNA sponges act as miRNA inhibitors to attenuate pressure overload-induced cardiac hypertrophy. Mol Ther.

[259] Kawai M., Ibaraki H., Takashima Y., Kanazawa T., Okada H. (2021). Development of a liquid crystal formulation that can penetrate the stratum corneum for intradermal delivery of small interfering RNA. Mol Pharm.

[260] Sarepta Therapeutics. Sarepta therapeutics announces that FDA has lifted its clinical hold on SRP-5051 for the treatment of duchenne muscular dystrophy. Available from: https://investorrelations.sarepta.com/news-releases/news-release-details/sarepta-therapeutics-announces-fda-has-lifted-its-clinical-hold. (Accessed 9 June 2022).

[261] Tang Y., Wang G. (2021). NIR light-responsive nanocarriers for controlled release. J Photochem Photobiol, C.

[262] Castro F., Martins C., Silveira M.J., Moura R.P., Pereira C.L., Sarmento B. (2021). Advances on erythrocyte-mimicking nanovehicles to overcome barriers in biological microenvironments. Adv Drug Deliv Rev.

[263] Solanki R., Bhatia D. (2024). Stimulus-responsive hydrogels for targeted cancer therapy. Gels.

[264] Yu H., Gao R., Liu Y., Fu L., Zhou J., Li L. (2024). Stimulus-responsive hydrogels as drug delivery systems for inflammation targeted therapy. Adv Sci (Weinh).

[265] Zhou H., Zhang S., Liu X., Feng A., Chen S., Liu W. (2025). Microneedle delivery platform integrated with staphylococcus epidermidis-derived extracellular vesicles-based nanoantibiotics for efficient bacterial infection atopic dermatitis treatment. Acta Pharm Sin B.

[266] Yang B., Wang H., Kong J., Fang X. (2024). Long-term monitoring of ultratrace nucleic acids using tetrahedral nanostructure-based NgAgo on wearable microneedles. Nat Commun.

[267] Qin Y., Cui F., Lu Y., Yang P., Gou W., Tang Z. (2025). Toward precision medicine: End-to-end design and construction of integrated microneedle-based theranostic systems. J Control Release.

[268] Zlatev I., Castoreno A., Brown C.R., Qin J., Waldron S., Schlegel M.K. (2018). Reversal of siRNA-mediated gene silencing. in vivo. Nat Biotechnol.

[269] In 't Veld S.G.J.G., Arkani M., Post E., Antunes-Ferreira M., D'Ambrosi S., Vessies D.C.L. (2022). Detection and localization of early- and late-stage cancers using platelet RNA. Cancer Cell.

[270] Reggiardo R.E., Maroli S.V., Peddu V., Davidson A.E., Hill A., LaMontagne E. (2023). Profiling of repetitive RNA sequences in the blood plasma of patients with cancer. Nat Biomed Eng.

[271] GlobalData Healthcare. Pay-for-performance deals for pharmaceuticals continue to gain momentum in the US. Available from: https://www.pharmaceutical-technology.com/comment/commentpay-for-performance-deals-for-pharmaceuticals-continue-to-gain-momentum-in-the-us-5755103/. (Accessed 3 March 2017).

[272] Li C., Callahan A.J., Simon M.D., Totaro K.A., Mijalis A.J., Phadke K.S. (2021). Fully automated fast-flow synthesis of antisense phosphorodiamidate morpholino oligomers. Nat Commun.

[273] Nakajima S., Nakamizo S., Nomura T., Ishida Y., Sawada Y., Kabashima K. (2024). Integrating multi-omics approaches in deciphering atopic dermatitis pathogenesis and future therapeutic directions. Allergy.

[274] Quílez C., Bebiano L.B., Jones E., Maver U., Meesters L., Parzymies P. (2024). Targeting the complexity of *in vitro* skin models: a review of cutting-edge developments. J Invest Dermatol.

[275] Bottari F., Daems E., de Vries A.M., Van Wielendaele P., Trashin S., Blust R. (2020). Do aptamers always bind? The need for a multifaceted analytical approach when demonstrating binding affinity between aptamer and low molecular weight compounds. J Am Chem Soc.

[276] Zhao Y., Yavari K., Liu J. (2022). Critical evaluation of aptamer binding for biosensor designs. Trends Analyt Chem.

[277] Yu A.M., Tu M.J. (2022). Deliver the promise: RNAs as a new class of molecular entities for therapy and vaccination. Pharmacol Ther.

[278] Charlesworth C.T., Deshpande P.S., Dever D.P., Camarena J., Lemgart V.T., Cromer M.K. (2019). Identification of preexisting adaptive immunity to Cas9 proteins in humans. Nat Med.

[279] Abudayyeh O.O., Gootenberg J.S., Essletzbichler P., Han S., Joung J., Belanto J.J. (2017). RNA targeting with CRISPR-Cas13a. Nature.

